# Layer-Scale and Chip-Scale Transfer Techniques for Functional Devices and Systems: A Review

**DOI:** 10.3390/nano11040842

**Published:** 2021-03-25

**Authors:** Zheng Gong

**Affiliations:** 1 Institute of Semiconductors, Guangdong Academy of Sciences, No. 363 Changxing Road, Tianhe District, Guangzhou 510650, China; zheng_gong@gdisit.com; 2 Foshan Debao Display Technology Co Ltd., Room 508-1, Level 5, Block A, Golden Valley Optoelectronics, Nanhai District, Foshan 528200, China

**Keywords:** layer transfer, chip transfer, hetero-integration, micro-LED displays, flexible electronics

## Abstract

Hetero-integration of functional semiconductor layers and devices has received strong research interest from both academia and industry. While conventional techniques such as pick-and-place and wafer bonding can partially address this challenge, a variety of new layer transfer and chip-scale transfer technologies have been developed. In this review, we summarize such transfer techniques for heterogeneous integration of ultrathin semiconductor layers or chips to a receiving substrate for many applications, such as microdisplays and flexible electronics. We showed that a wide range of materials, devices, and systems with expanded functionalities and improved performance can be demonstrated by using these technologies. Finally, we give a detailed analysis of the advantages and disadvantages of these techniques, and discuss the future research directions of layer transfer and chip transfer techniques.

## 1. Introduction

Semiconductor materials and devices are the building blocks for most of modern electronics and integration circuits. From individual LEDs to large TVs, smart cars, and computers, needless to say, we can find semiconductors almost everywhere. They have had a profound impact on our human daily lives. Advancement in epitaxial technology has led to the formation of high-quality semiconductor layers on particular substrates. These semiconductor layers can then be turned into functional devices using conventional lithography and microfabrication processes, such as LEDs, lasers, sensors, transistors, etc. These devices can then be diced and packaged in a manner suited for later assembly and for demonstrating diverse functional systems and apparatus.

While, in many cases, the growth substrate is preserved after the chip fabrication, there are many occasions where the semiconductor layers need to be very thin (from nm to µm scale) and transferred to a different substrate by removing the growth substrate [1,2,3,4,5,6,7,8,9,10,11,12,13,14,15,16,17,18,19,20,21,22,23,24,25,26,27,28,29,30,31,32,33,34,35,36,37,38,39,40,41,42,43,44,45,46,47,48,49,50,51,52,53,54,55,56,57,58,59,60,61,62,63,64,65,66,67,68,69,70,71,72,73,74,75,76,77,78,79,80,81], termed as “layer transfer” in the following. One example of such requirements is the high-power GaN LED chips used for general lighting purposes [82,83,84]. In order to deliver high power for lighting, these LEDs must be very efficient and sustain high current operation, which implies these LEDs must have a very good thermal dissipation capability. The GaN LED layers are commonly grown on sapphire substrates, which, however, are not good enough for heat dissipation. Therefore, for high-power LED chips, the LED layer has to be transferred and bonded to a substrate which is more thermally conductive, for instance, Cu [83]. An additional benefit of the thin LED after substrate removal is boosting of the light extraction efficiency (LEE) [85,86]. It is well known that thick sapphire can cause light absorption and total internal reflection, such that part of the light is trapped inside the LED chip, resulting in limited LEE [86]. While some thinning techniques [87,88,89,90], such as mechanical grinding, polishing, chemical wet etching, dry etching, etc., are available for fabricating very thin III-V semiconductor layers or devices grown on GaAs and Si, they are generally more difficult for III-nitride layers grown on sapphire and SiC. Sapphire and SiC are hard substrate materials which are almost inert to most of etchants. Thinning down of these materials to 100 µm is possible but complete removal of the substrate is not realistic by thinning technology.

Chip scale transfer is another direction which has received strong interest from both academia and industry [1,91,92,93,94,95,96,97,98,99,100,101,102,103,104,105,106]. This is primarily driven by Moore’s law, and the requirement of integrating more functional chips onto one single substrate. Different from the layer transfer discussed earlier, the chip to be transferred here is much smaller in size. Conventional chip-scale assembly is based on robotic pick-and-place, or flip-chip bonding [82,105,107,108,109,110,111,112,113,114,115,116,117,118,119,120,121]. However, with shrinking of the die size, pick-and-place techniques lose their assembly efficiency and accuracy. For devices with dimensions less than 100 µm, adhesive capillary forces are often bigger than gravitational forces [122,123]. As a result, releasing the devices from the robot becomes difficult. Therefore, there is growing interest in developing chip-scale assembly techniques with high assembly speed, high yield, and good placement accuracy. The recently emerged micro-LED display technology, for instance, is one of the major driving forces for small chip transfer [98,99,100,105,124,125,126,127,128,129,130,131,132,133,134,135]. Being small, self-emissive inorganic semiconductor devices, micro-LED displays have a number of distinct advantages over conventional LCD and OLED techniques, such as higher brightness, lower power consumption, faster switching, higher contrast, etc. Thanks to the intensive effort from both academy and industry, micro-LED displays have undergone a very fast development, however, the reduced pixel size causes serious challenges for micro-LED assembly and integration. In order to display images and break down the cost, the micro-LED must be accurately and quickly integrated onto the backplane. Taking a 4K display as an example, 25 million pixels would be required to be assembled onto the driver backplane. This is not realistic to be achieved using existing pick-and-place methods, considering that the transfer speed of a state-of-the-art surface-mounting-technology (SMT) machine is only 30k per hour. Therefore, there is strong interest in the industry to develop various mass-transfer techniques for fast and cost-effective assembly of ultrasmall chips.

The development of flexible or wearable devices is another major factor driving the need for developing cost-effective layer transfer and chip transfer techniques [124,129,136,137,138,139,140,141,142,143,144]. Flexible electronics can find a wide range of applications, such as flexible or stretchable displays [137,145,146,147,148,149,150,151,152,153], flexible transistors [154,155,156,157,158,159,160], flexible solar cells [77,92,161], flexible sensors [162,163,164,165,166], wearable medical devices [127,167,168,169], and human–machine interfaces [170,171,172,173,174]. While organic semiconductors are naturally suited for fabricating flexible devices because of their solution processable and conformal coating compatibility with the flexible substrate, they commonly show compromised device performance, compared with the inorganic counterparts. Inorganic semiconductors, on the other hand, commonly have much better performances in terms of the electron mobility, stability, and lifetime, but they are not able to be grown on plastic substrates directly. Another reason is that the processing conditions of inorganic semiconductors are not compatible with the flexible substrate. This hinders the direct fabrication of inorganic devices on a plastic substrate, because the processing temperature is much higher than the flexible plastic can withstand. Therefore, separating the inorganic device fabrication from its assembly process onto the flexible substrate is more realistic. A variety of layer (or chip) transfer techniques for inorganic semiconductors have evolved for this purpose.

Finally, heterogeneous integration of multiple layers or chip components onto one single substrate is also one major driving force for developing layer and chip transfer techniques [106,175,176,177]. The layer and chip transfer techniques allow the assembly of hybrid devices with expanded functionalities that could not be otherwise realized by using individual devices. In some cases, 3D integration can be even enabled [178,179]. While there are existing techniques such as pick-and-place or microassembly using robots for heterogeneous integration of various components, these methods suffer from low assembly efficiency. Some parallel layer transfer techniques are being developed to address this challenge.

In this review, we first introduce various layer transfer techniques for heterogeneous integration of ultrathin semiconductor layers onto a targeting substrate. These techniques provide a strong foundation for heterogeneous integration of dissimilar materials, which could expand the functionality of one particular device to multiple types of devices on a single wafer. We then review the chip-scale transfer techniques evolved for some particular applications such as microdisplays and flexible electronics. We also explore future multifunctional systems that could be realized by layer transfer and chip transfer approaches. Finally, we give a summary of key outcomes from this review and outlook in the future.

## 2. Layer Transfer Techniques

Layer transfer is a technique to transfer a layer of a particular semiconductor material, often of a wafer-scale size, from the original substrate to the target substrate of interest. The key process is to remove the growth substrate on which the semiconductor layer is deposited. The technique allows the integration of both lattice-matched and mismatched material systems for enabling extended functionality and performance by assembling diverse materials or devices in a more compact space. The additional benefit is the potential reuse of the growth substrate if it is not damaged during lift-off, thereby reducing the cost [3,4,15]. As one example, the transfer of GaN micro-LEDs onto silicon complementary metal oxide semiconductor (CMOS) allows a high-quality display with additional functionality such as pulse control.

**Figure 1:**
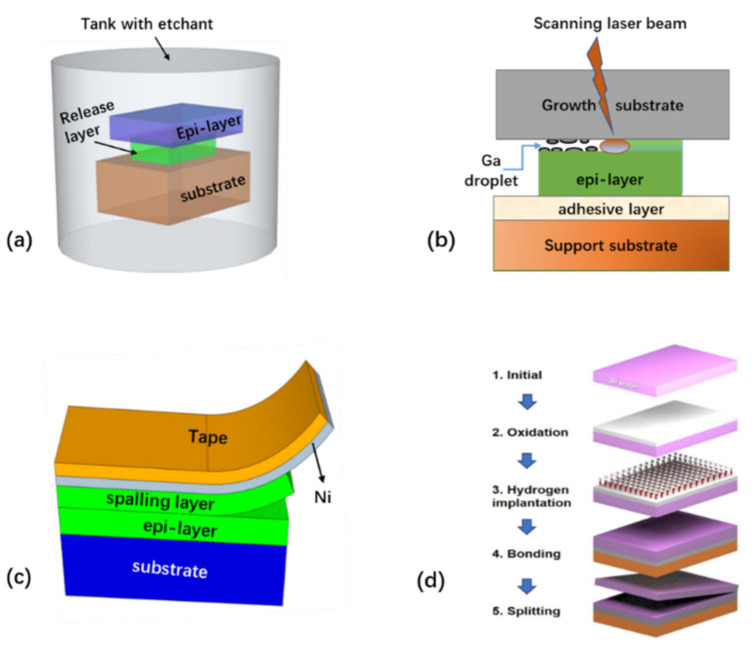
Sketches of common layer transfer techniques: (**a**) chemical epitaxial lift-off, (**b**) laser lift-off, (**c**) mechanical spalling, and (**d**) ion cutting.

The conventional method for layer transfer is mainly based on wafer bonding and mechanical thinning [87,88,89,90]. However, thinning techniques are difficult for accurate control of the film thickness and surface roughness across the wafer. For instance, in most cases, reducing the layer thickness down to 10 µm by mechanical thinning is extremely challenging. To address these challenges, a variety of new lift-off technologies have been developed to assist the wafer-scale layer transfer, some of which have the potential for volume production. These include epitaxial lift-off (ELO), mechanical spalling, laser lift-off, and ion cutting, as schematically shown in Figure 1 below.

### 2.1. Layer Transfer by Epitaxial Lift-off (ELO)

Referring to Figure 1a, ELO relies on the removal of a releasable or sacrificial layer introduced in the epi-stacks using various chemical etchants, such that the epi-layer on top of the releasing layer can be transferred to other substrates while preserving the original growth substrate [1,6,7,8,10,11,12,13,14,15,16,17,18,19,20,21,22,23,24,25,26,27,28,29,30,31,32,33,34,35,37,38,39,40,41,42,43,44,45,47,48,50,51,52,53,54,55,56,57,58,59,60,61,62,63,64,65,66,67]. The primary requirements for this technique are: (i) high etch selectivity of the releasing layer, (ii) the capability for high-quality growth of the epi-layer on the releasing layer, and (iii) minimized damage to the epi-layer after release. Therefore, the suitable release layer not only determines the epi-layer quality, but also determines the ELO quality. Selecting the right release layer is highly dependent on the epi-layer (to be released), substrate, and etchant solvent to be used. Commonly, chemical lift-off of small samples is relatively quick, but wafer-level release remains challenging. Depending on the specific release layer in the epi-stack, the etching duration for releasing the full wafer may vary from a few hours to a few days, which may impose practical limitations for large-volume production. Therefore, to accelerate the release, several variants of the conventional ELO have been proposed, including weight-assisted, surface tension force-assisted, or roller-assisted ELO techniques [15,53,54].

#### 2.1.1. ELO Assisted by Lattice-Matched Release Layer

**Figure 2:**
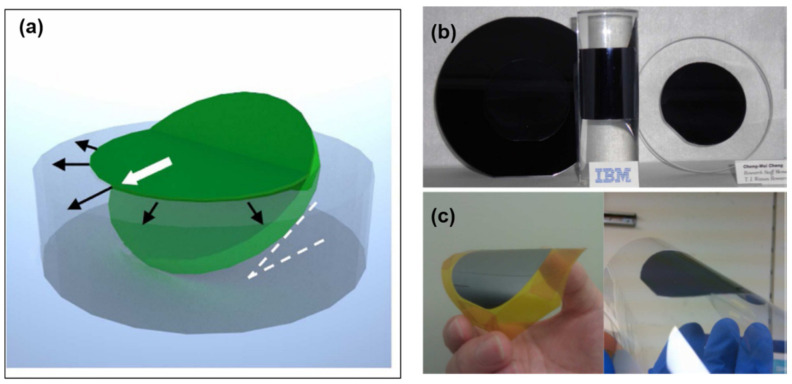
(**a**) Sketch of surface tension-assisted epitaxial lift-off (ELO) transfer techniques. (**b**) Demonstrations of the transferred GaAs thin films to the rigid substrate (left image, GaAs on 4′’ Si wafer. Center image, GaAs on curved solid object. Right image, GaAs on glass) and (**c**) flexible substrates (left, GaAs on tape. Right, GaAs on flexible sheet). Adapted from [15].

Earlier ELO studies of III-V semiconductor layers in the past were mainly based on lattice-matched release layers, primarily because the growth of high-quality layered semiconductors on a lattice-matched release layer is much easier than on a dissimilar sacrificial layer. Depending on the specific epitaxial structures and the growth substrate, the release layer and corresponding etching solvent can be quite different. For example, one common release layer for III-V semiconductors grown on GaAs is AlAs [57], which is a material lattice- matched with the substrate and can be removed by hydrofluoric acid. However, a recent investigation reveals that etching AlAs using hydrofluoric acid is fast, but it leads to reaction residuals and increased roughness of the released layer and substrate [15]. To alleviate this issue, lattice-matched AlInP was introduced to act as the release layer, which can be etched by a different solvent, hydrochloride acid [15]. In the latter case, very smooth III-V layers and substrate free from residuals can be achieved via a modified ELO technique termed as “surface tension-assisted ELO”, enabling the prospect of substrate reuse (Figure 2). In the case of InP-based nanomembranes grown on InP substrates, InGaAs was found to be a desirable sacrificial layer, which can be selectively etched by either HF+H_2_O_2_ [60] or H_3_PO_4_ and H_2_O_2_ [9]. Alternatively, InAlAs was also explored for releasing InP-based devices, which has higher etch selectivity, and less dependence on the crystal orientation caused by the etching solvent, compared with InGaAs [47]. In all cases, the release layer and corresponding etching solvent are chosen such that the semiconductor layer to be released maintains high epitaxy quality while the etch selectivity is high.

Releasing III-V semiconductor layers from the growth substrate to a receiving substrate by ELO has been researched for many years, and can be dated back to 1978. The ELO technique based on a lattice-matched release layer is now well developed, particularly for high-efficiency III-V solar cells [5,18,36,46].

#### 2.1.2. ELO Assisted by Heterogeneous Release Layer

More recently, dissimilar release layers have also been explored, particularlyfor III-nitride layer release. Similar to III-V semiconductors, releasing III-nitride by ELO is also possible, but more challenging. Unlike III-V semiconductors, III-nitride layers themselves are resistant to most of etchant solvents and, therefore, they are not ideal release layers for GaN. To overcome this limit, most of efforts are therefore focused on introducing a dissimilar release layer rather than GaN alloy into the III-nitride epi-stack for ELO [16,19,29,31,41,42,44,45,52,62]. However, the epitaxial growth of GaN on a heterogeneous release layer is more challenging, and may lead to degraded material quality due to the lattice mismatch. Despite of these challenges, various release layers, including SiO_2_ [16,19,62], Ga_2_O_3_ [31], CrN [29], Nb_2_N [45], AlN [41,42], and ZnO [44,52], have been successfully explored to lift off GaN membranes.

Hsueh et al. demonstrated the use of ZnO as a sacrificial layer [52]. A 2-inch ZnO template layer was grown on a sapphire substrate by using pulsed laser deposition (PLD). The wafer was then loaded into an hydride organometallic vapor phase epitaxy (HOVPE) chamber for further growth of GaN epi-layers on top of the ZnO release layer. A low-pressure/temperature HOVPE approach was adopted to prevent ZnO decomposition. The completed wafer was then fixed onto a glass support using wax. Afterward, ELO was performed using HCL as an etchant solvent, resulting in the entire transfer of the 2-inch GaN epi-layer to the support substrate without apparent degradation of the GaN epi-layer. Due to the lateral etching mechanism, the etch rate at the wafer edge was found to be faster than in the wafer center. The surface of the released substrate was found to be very smooth, opening up the prospect of substrate reuse and cost reduction.

As another example of suitable release layers for GaN lift-off, CrN [29] was formed on a sapphire substrate by depositing chromium with a radio frequency (RF) sputtering system, followed by a nitridation process. LED layer structures were then grown on the CrN buffer/substrate by low-pressure HOVPE. A gold layer was electroplated to the p-GaN side to act as the support substrate. By selectively etching the CrN release layer using a mixture of H_2_O, Ce(NH4)_2_ (NO_3_)_6_, and HClO_4_, high-performance vertical LEDs transferred onto a gold support can be achieved. It was found that such vertical LEDs have much smaller serial resistance, showing the potential for general lighting. However, in this particular work, only centimeter-scale lift-off was realized. Whether it is suited for wafer-level ELO remains an open question.

Hsueh et al. demonstrated the use of Ga_2_O_3_ [31] as a release layer for III-nitride lift-off. LED layers grown on Ga_2_O_3_ can then be readily removed by HF, resulting in the transfer of 2-inch GaN LEDs to the electroplated Cu support. As GaN is inert to HF etching, the LED layer experiences minimal damage. One disadvantage of Ga_2_O_3_ is its decomposition under high temperatures in a H_2_-rich environment. Therefore, Ga_2_O_3_ must be grown separately in a N-rich atmosphere.

While various dissimilar release layer-assisted ELO techniques have been explored, great challenges remain for releasing III-nitride semiconductors. Thus far, the feasibility for commercial production of nitride compound semiconductors based on release layer-assisted ELO is not proven yet. The major challenges are relevant to the compromised epitaxy quality grown on a dissimilar sacrificial layer, and the etching-induced damage to the semiconductor layer to be released.

#### 2.1.3. ELO Assisted by Micro/Nanopatterned Structures

In some cases, micro/nanopatterned structures can be used to assist the layer transfer [16]. In one example, nanoporous SiO_2_ [62] is formed by using an anodized alumina template as a mask. A GaN epi-layer is then grown on top of the nanoporous SiO_2_. After finishing the growth, wet etching of the nanoporous SiO_2_ using HF is performed, leading to the spontaneous release of the GaN film. The nanoporous SiO_2_ also facilitates the lateral epitaxial growth of high-quality GaN on the nanoporous SiO_2_, which can reduce the dislocation density in the epi-layer.

**Figure 3:**
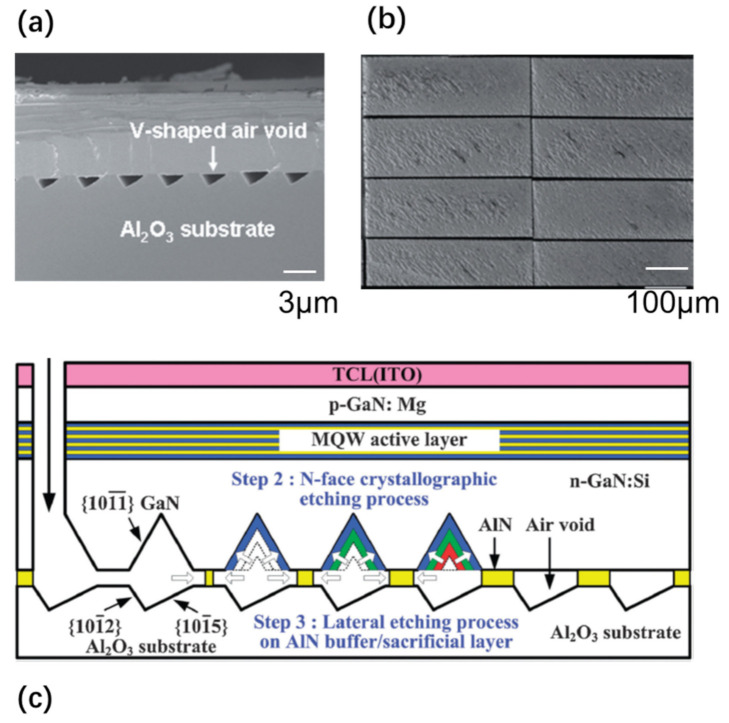
(**a**) An LED epitaxial layer was grown on the patterned sapphire substrate with V-shaped void channels. (**b**) The individual LED chips defined through the laser scribing process were lifted off from the void structures. (**c**) Schematic diagram of the multiple-quantum-well (MQW) LED structure topped with a transparent conductive layer (TCL) ITO for the chemical lift-off (CLO) process. Adapted with permission of [42]. Copyright Applied Physics Express, 2010.

Void microstructures are also utilized to assist the layer release [17,42]. Lin et al. [42] demonstrated the growth of nitride semiconductors on a truncated triangle striped pattern sapphire substrate (Figure 3). This leads to the formation of an epi-stack with embedded void structures. These voids facilitate the wet etching of a thin AlN sacrificial layer in the lateral direction by hot KOH etching, leading to the formation of released GaN layers.

#### 2.1.4. Photoelectrochemical (PEC) or Electrochemical (EC) Etching

**Figure 4:**
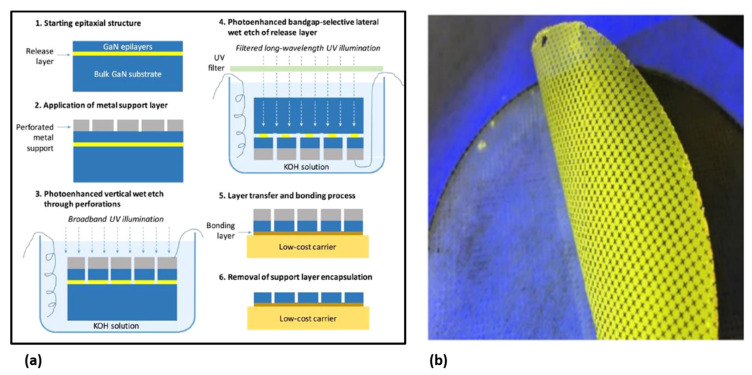
(**a**) The detailed process flow of ELO based on photoelectrochemical (PEC) etching, and (**b**) full 4-inch GaN layer released by PEC etching. Adapted with permission from [61]. Copyright John Wiley and Sons, 2017.

PEC etching methods [1,6,8,23,30,34,43,48,50,59,61,65] have been developed to release III-nitride, although conventional wet etching is difficult for etching III-nitride. This method exploits the illumination that only absorbs in the specific layer, in order to form electron–hole pairs in the semiconductor material [61]. The photogenerated holes result in the oxidation and dissolution of the semiconductor layer, while the electrons are moved to the cathode to participate in a reduction reaction. An example of PEC etching is demonstrated by Youtsey et al. [61]. The detailed PEC etching procedure is shown in Figure 4a. By selective PEC etching of an InGaN release layer, wafer-scale lift-off of 4-inch GaN films was demonstrated (Figure 4b).

In 2011, Lin et al. reported the lift-off of InGaN LED structures using a hybrid approach of PEC etching and mechanical peeling [43]. LED structures are prepatterned and fabricated on a sapphire substrate, followed by PEC lateral etching of the InGaN/GaN superlattices. A tape is then laminated onto the LED top. Mechanical peeling of the tape allows LEDs to be successfully transferred to the tape, with the emission blueshift to a shorter wavelength due to the strain relaxation. In a more recent work [8], similar PEC etching is conducted to release nanopillar LEDs defined by nanosphere lithography.

One of the disadvantages of PEC etching methods, however, is the requirement of external illumination sources. To address this issue, EC etching techniques without light illumination have also bene developed. For instance, Park et al. developed a method based on dope-selective EC etching to release GaN membranes [48]. The EC etching is fast for n-GaN but it is almost inert for p-GaN and undoped GaN. Making use of this highly selective etching, successful lift-off of patterned p-GaN films was achieved.

**Figure 5:**
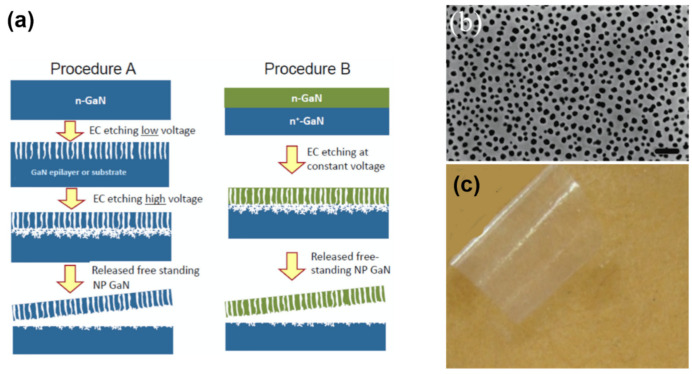
(**a**) Sketches of two different EC etching procedures, (**b**) SEM image of the porous structures formed by EC etching, and (**c**) the GaN membrane released from the porous GaN layer formed by EC etching. NP GaN in (a) stands for the nanoporous GaN. Adapted with permission from [66]. Copyright IOP Publishing, 2010.

Modified EC etching methods for GaN release have also been developed [66]. Porous GaN formed by EC etching is exploited to assist the lift-off, whose porosity can be tuned by changing the doping concentration and adjusting the etching voltage. Zhang et al. [66] developed two different schemes for GaN layer transfer (Figure 5). In the first procedure (i.e, Procedure A shown in Figure 5a), two-stage EC etching was applied to n-doped GaN. Initially, a lower bias is applied to the GaN, and results in the formation of a porous GaN film of a certain depth. The bias is then increased in the second stage, leading to the formation of a void layer with larger porosity exactly below the porous layer generated in the first stage. Consequently, the GaN film can be released from the substrate. Alternatively, GaN release is also demonstrated based on a GaN sample with lightly doped n-GaN and heavily doped n-GaN, but only a constant bias is applied for conducting EC etching (Procedure B in Figure 5b). In this case, a void layer with larger porosity can be formed below lightly doped GaN. In both cases, centimeter-scale free-standing GaN membranes without degradation have been achieved. In the latter case, the thickness of the transferred layer can be accurately controlled by the doping concentration. However, wafer-scale release of GaN based on EC etching remains challenging.

In the above, successful lift-off of a single doped GaN layer from porous GaN formed by EC etching is demonstrated. This idea can be further extended to release InGaN/GaN MQW LED structures overgrown on a porous-GaN template formed by EC etching [64]. PEC- or EC-based transfer techniques have some advantages, compared with other ELO methods. Since the release layer is a GaN-based material (e.g., InGaN), the epi-layer quality can be maintained, and only one metal-organic chemical vapor deposition (MOCVD) growth cycle is needed, without introducing an extra dissimilar release layer which is commonly deposited by different equipment. One potential disadvantage, however, is the high surface roughness after lift-off. Furthermore, large-scale lift-off based on these techniques remains challenging.

### 2.2. Layer Transfer by Laser Lift-Off (LLO)

While ELO discussed in the previous section has achieved good success in some particular cases, the nature of wet etching also tends to cause partial damage to the semiconductor layer to be released. Finding a chemical etchant which is absolutely inert to the semiconductor layer but has a very high etch rate for the sacrificial layer is very difficult. The long etch duration for the wafer-level of ELO in many cases is also another constraint for fast production. Furthermore, wet etching is commonly not environmentally friendly, and is also hazardous in many cases. For these reasons, a few “dry” lift-off mechanisms are explored for layer transfer to minimize wet etching-induced damage and accelerate the lift-off.

One example of dry lift-off for layer release is LLO [180,181,182,183,184,185,186,187]. As schematically shown in Figure 1b, LLO makes use of the difference in absorption of the laser light between the substrate and the layer being released. In the case of GaN LEDs grown on a sapphire substrate, for example, the GaN epi-layer has a band gap of about 3.3 eV, whereas the sapphire band gap energy is ~ 9.9 eV. Short-wavelength laser light is therefore transparent for the sapphire, and but strongly absorbed in the GaN layer, thereby generating intense heat. This localized heat leads to the decomposition of the GaN near to the GaN/sapphire interface into Ga droplets and nitrogen gas, thereby separating the epi-layer from the substrate.

**Figure 6:**
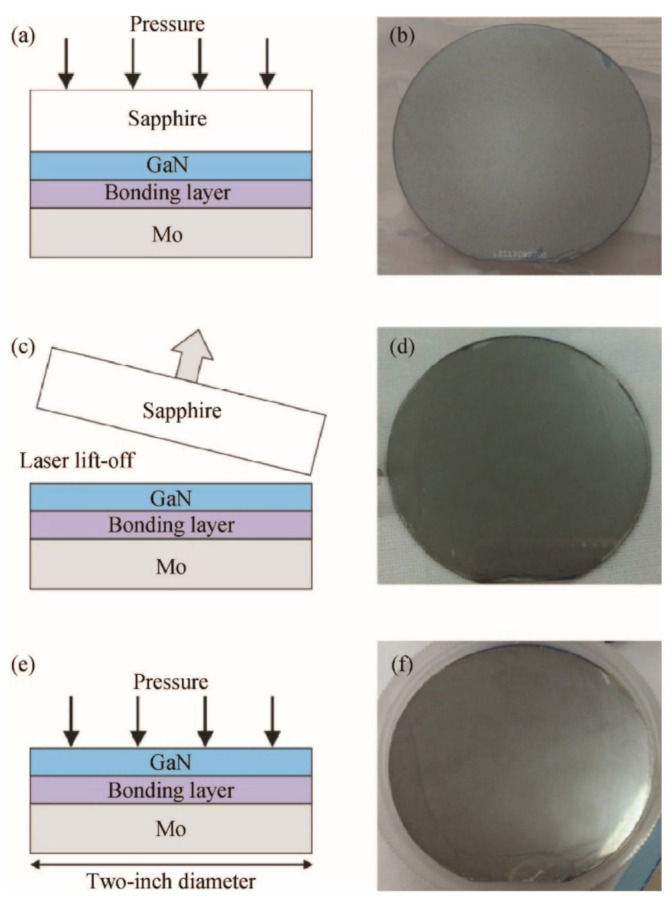
Schematic diagram of sample (**a**) after first bonding, (**c**) after laser-lift-off, and (**e**) after second bonding; image of the 2-inch-diameter sample after it went through (**b**) first bonding, (**d**) laser-lift-off, and (**f**) second bonding. Adapted with permission from [181]. Copyright IOP Science, 2016.

One particular application of this technique is the wafer-scale layer transfer of a GaN thin film to a support substrate. To assist the laser lift-off, the wafer is commonly fixed onto a temporary substrate by wafer bonding or adhesive bonding. One example [181] of such a strategy is demonstrated by Wang et al. (Figure 6). GaN wafer grown on sapphire was bonded with a Mo substrate using Ni/Au as the bonding layer. LLO was conducted to take off the sapphire substrate. A further bonding and subsequent annealing were applied to the released GaN layer on the Mo substrate. As a result, the resulting two-inch-diameter GaN template showed improved stability and a minimized stress state. Similarly, successful thin-layer transfer of 2-inch GaN via LLO has also been achieved by other support substrates including GaAs and polydimethylsiloxane (PDMS) [183].

Many investigations indicate that LLO can also be used for fabricating free-standing GaN substrates with large thickness [180,183,185,186]. The free-standing GaN wafer size by LLO was limited to 1.5~2 inches in earlier investigations [185,186], but 4-inch free-standing GaN wafers have been demonstrated recently [180]. Major factors preventing the achievement of large, thick GaN templates include cracks induced by the thermal strain relaxation and laser-induced shock waves, causing damage at the N-polar face of GaN. It was reported that a heating plate above 800 degrees is helpful to release the compressive strain and avoid cracks during LLO. Laser spot size is another critical parameter affecting the laser-induced damage [180].

**Figure 7:**
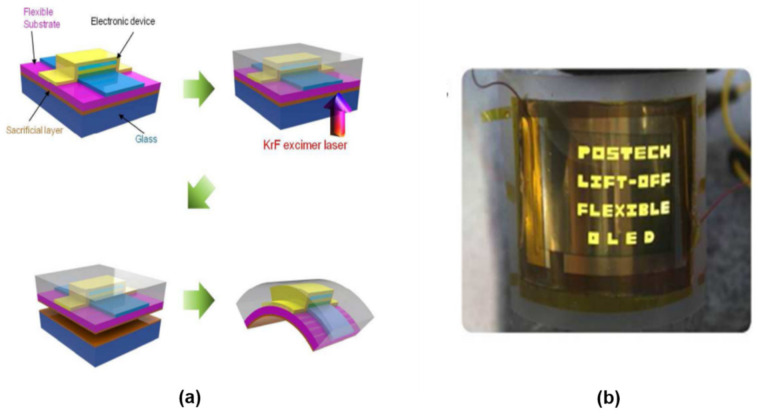
(**a**) Schematic diagram of the process flow for fabricating flexible OLED display by LLO. (**b**) The corresponding flexible OLED display fabricated by laser lift-off (LLO) based on the technology shown in (**a**). A 248 nm excimer KrF laser with a pulse width of 25 ns is used for the LLO. Adapted with permissions from [188]. Copyright Royal Society of Chemistry, 2014.

The laser lift-off technique is also applicable for fabricating flexible devices [124,182,184,188,189,190]. An example of the process flow for making flexible OLED displays based on LLO is shown in Figure 7. A sacrificial layer of polyimide [184] or α-GaOx [188] is formed on glass substrate. OLED devices are then formed on the sacrificial layer. Laser beam scanning results in intense heat generated in the interface between the sacrificial layer and glass substrate. Consequently, the sacrificial layer is ablated, resulting in the top OLEDs becoming delaminated from the substrate. This technique is now applied in large-volume production of flexible OLED display screens [184].

The LLO method is fast and scalable for any wafer size. For example, a 2” wafer, in principle, could be lifted off in a few seconds. However, in order to achieve high-quality transferred layers free from damage by laser lift-off, the beam quality and control must be well controlled. The cost of LLO facilities is another disadvantage that restricts its availability to regular users.

### 2.3. Layer Transfer by Mechanical Release

Mechanical release relies on mechanical force to separate the semiconductor layer from a growth substrate and transfer it to a support substrate. Broadly speaking, there are three major mechanical release approaches: spalling, 2D layer assisted peeling, and water-induced de-lamination.

#### 2.3.1. Stress-Induced Delamination

**Figure 8:**
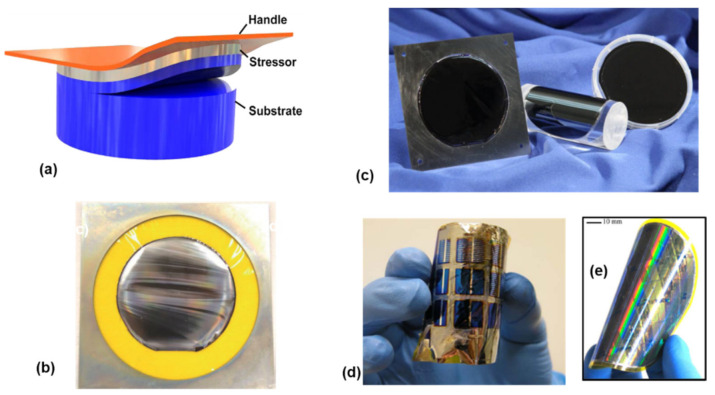
(**a**) Sketch of the mechanical spalling. Adapted from [74]. (**b**) Optical image of the released GaN film. Adapted with permission from [73]. Copyright IOP Science, 2013. (**c**) Optical images of Si on plastic mounted in a handling frame, 8 µm thick III–V multijunction layers on tape and mounted on a cylinder, and the bulk Si substrate from which the 20 µm thick layer was removed (from left to right). (**d**) Solar cells. Adapted with permission from [71]. Copyright IEEE, 2016. (**e**) flexible CMOS circuits fabricated by spalling techniques. Adapted with permission from [76]. Copyright American Chemical Society, 2012.

Stress-induced delamination, or spalling [70,71,72,73,74,75,76,77,79,80,81], refers to a phenomenon where a layer with tensile stress tends to peel away from the substrate where the layer is grown, accompanied by the removal of a portion of the substrate material (Figure 8a). The mechanism behind spalling (or cracking) is due to the edge load created by the tensile stressor which guides the crack to be propagated at an equilibrium depth below the interface [71,73].

To achieve a controllable fracture and continuous film transfer, a tensile stressor layer with suitable thickness is coated on the substrate, followed by attaching a tape as the handle layer. A small force is then applied on the handle layer, resulting in forming a crack at a predetermined depth in the substrate. By mechanically guiding the handle layer, this crack can be guided and propagated in a controllable manner, resulting in transferring a portion of the material from the substrate to the handle layer [74].

This effect has been known for many years, and now it is possible to make use of this effect to achieve wafer-scale layer transfer of a variety of materials and devices, as shown in Figure 8. For example, silicon [70,75], InGaP/(In)GaAs [77], and GaN [73,80] have been successfully released by spalling. A wide range of flexible devices have also been demonstrated by spalling, including solar cells [71,72,77], LEDs [73,80], CMOS [74,76,81], etc. The figure below shows, for example, representative images of the full-wafer scale semiconductor layers and devices transferred by using this technique (Figure 8b–e).

Compared with epitaxial lift-off, stress-controlled spalling is much simpler, independent of area, and does not require the use of specialized etch layers. Substrate reuse is also demonstrated, opening up the prospect of cost reduction. One disadvantage of this technique, however, is fracture depth (or the thickness of the transferred layer) control, which is largely dependent on a variety of factors such as the stress amplitude, stress layer thickness, the stress layer material, and also the substrate material [74]. Accurate control of the thickness of the transferred layer induced by the spalling is therefore possible but extremely challenging. Another challenge lies in the residual stress and slight curvature that the layers possess after spalling. To process such thin, stressed films requires the development of particular film handling strategies and equipment. The third challenge lies in the high roughness of the released layer. For instance, roughnesses up to 500 nm root mean square (RMS) have been reported for released GaN [80]. The high roughness of the released layer is undesirable for subsequent device fabrication and integration.

#### 2.3.2. 2D Layer-Assisted Delamination

**Figure 9:**
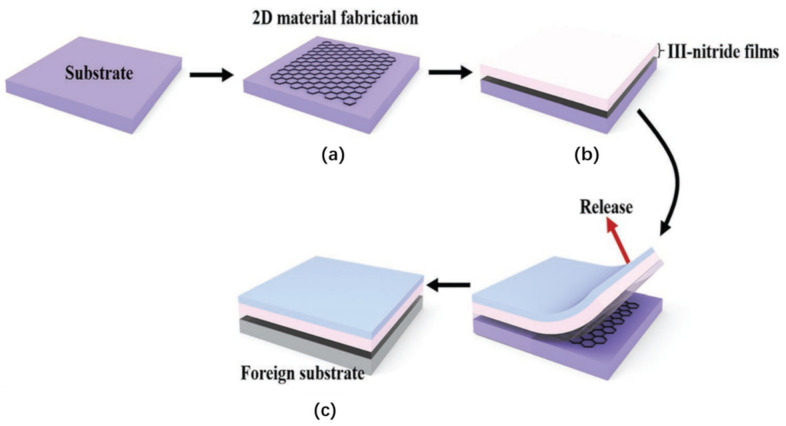
Schematic of 2D layer-assisted layer transfer, including three steps: (**a**) 2D material fabrication, (**b**) VDW epitaxy of III-nitride film, and (**c**) transfer printing onto foreign substrate. Adapted with permission from [207]. Copyright John Wiley and Sons, 2019.

2D layer-assisted delamination exploits the weak adhesion of the thin layer grown on layered 2D semiconductors [78,191,192,193,194,195,196,197,198,199,200,201,202,203,204,205,206,207]. This technique is also referred to as van der Waals (VDW) epitaxy [207] (Figure 9). Applying a mechanical force will break up the weak adhesion, and induce the delamination of the thin film from the 2D layered semiconductor. This method can potentially be used to obtain wafer-scale layer transfer at a low cost. Thanks to the advancement in epitaxial growth, VDW growth of high-quality III-nitride on such 2D semiconductor layers has been demonstrated, despite the large lattice mismatch. Various 2D layered materials, such as boron nitride [194,195,198,200] and graphene [78,191,192,193,196,197,199,201,202,203,204,205,206], have been explored to assist the lift-off of the thin semiconductor layers grown on 2D layered materials.

Kobayashi et al. demonstrated the high quality of growth AlGaN/GaN LED layers on a boron nitride single-crystal layer [194]. The boron nitride layer has two functions. Besides the role for subsequent lift-off, it also acts as the buffer layer for nucleation of the high-quality AlGaN/GaN LED layer. To prevent from formation of polycrystalline GaN, an AlGaN layer is first deposited on the BN layer, followed by growing the final GaN LED layer structures. Due to its weak adhesion on the boron nitride, the LED layer can then be readily separated from the substrate to an indium sheet by weak peeling. Based on similar techniques, prototype vertical LEDs have also been demonstrated by the same group [195]. However, only centimeter-scale layer transfer has been demonstrated. Wafer-scale transfer based on BN needs to be explored. To overcome the size limit, one possible route is to grow a BN monolayer on a modified substrate with a quasi-3D mainspring shape in a furnace tube, instead of the conventional flat substrate [200]. This allows a high-quality h-BN monolayer with a size up to 25 inch to be grown, which can be then transferred to sapphire substrate for growing GaN. A 2-inch GaN wafer free of misfit strain grown on a BN monolayer has been achieved based on this technique.

Graphene-assisted growth has also been explored for fabricating free-standing semiconductor membranes [78,197,199,201]. Since the nucleation of atoms on a pristine graphene surface is remarkably suppressed due to the inert surface reactivity of graphene, earlier studies were therefore mainly focused on growing 3D microstructures on graphene. For instance, Chung et al. demonstrated the growth of regular GaN microdisk arrays on graphene dot patterns using epitaxial lateral overgrowth (ELOG) [199]. In another example, GaN microrods on graphene were demonstrated [197].

**Figure 10:**
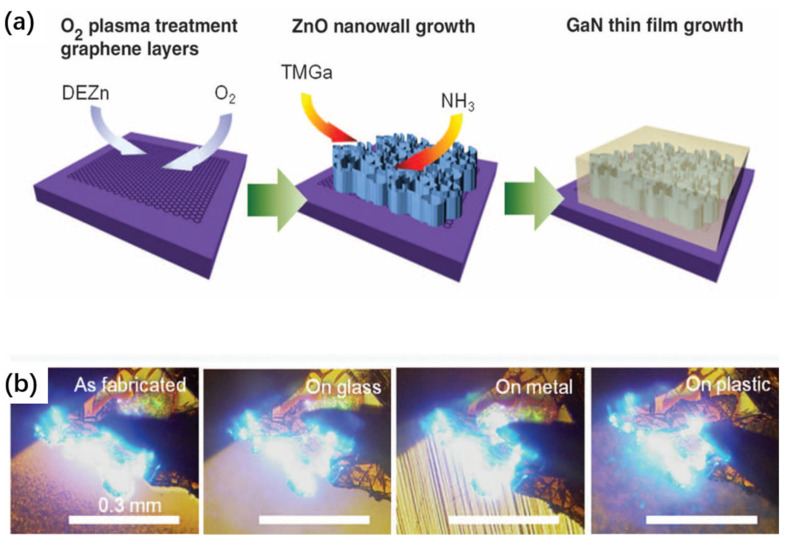
(**a**) Schematic illustrations of fabrication processes for epitaxial GaN thin films. (**b**) Optical images of light emissions from the as-fabricated LED on the original substrate and transferred LEDs on the foreign metal, glass, and plastic substrates. Adapted with permission from [191]. Copyright The American Association for the Advancement of Science, 2010.

The 2D nucleation difficulty, however, can be overcome by introducing an intermediate layer. For instance, Chung et al. [191] demonstrated that ZnO nanowalls grown on graphene can assist the subsequent growth of 2D GaN LED layers (Figure 10). Due to the same crystal structure and small lattice mismatch with GaN, epitaxial GaN films are formed on the nanowalls in a manner similar to the lateral overgrowth, and eventually a flat GaN overgrowth layer can be formed. Such high-quality GaN LED layers grown on graphene allow the fabrication of LEDs transferred to various substrates, including glass, metal, and plastic, by simple mechanical peeling, and strong blue emissions have been obtained from such devices.

In another study, to overcome the nucleation difficulty in 2D growth on graphene, an AlN buffer layer was introduced between the growth layer and graphene [202]. The graphene layer was grown on sapphire substrates by a catalyst-free atmospheric pressure chemical vapor deposition (APCVD) process, instead of using the complex transfer process of ex situ-grown graphene. An AlN buffer layer is then deposited on the nitrogen plasma-treated graphene to promote the GaN nucleation. The epitaxy is then finalized by growing GaN LED layers on the AlN/graphene buffer [202]. The APCVD method allows the high-quality growth of 2-inch single-crystal graphene. Due to the strain relaxation by the graphene, the as-prepared GaN shows significant improvements in the epitaxial quality, with a dislocation density as low as 1.7 × 10^7^cm^−2^. The fabricated LED devices therefore are able to deliver much high optical power output than the device directly grown on sapphire.

**Figure 11:**
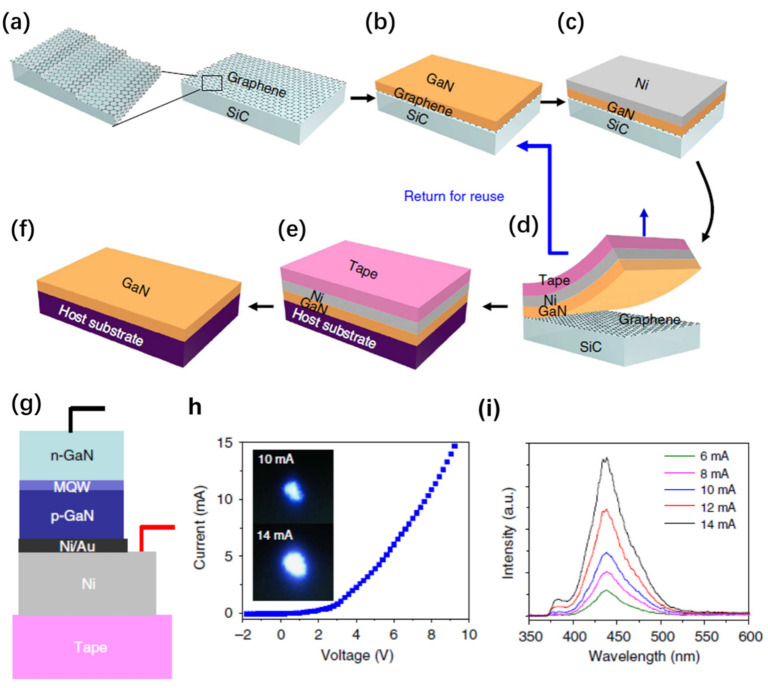
Schematic of a method for growing/transferring single-crystalline thin films on/from epitaxial graphene (**a**–**f**). (**g**) Schematic of a transferred visible LED device on the tape. (**h**) I–V characteristic of a transferred LED stack measured by applying positive bias on Ni and negative bias on n-GaN. The pictures of the LED emitting blue light are displayed in an inset. (**i**) Electroluminescence (EL) spectra of a transferred LED stack taken as a function of injection current. Adapted from [78].

Instead of using intermediate buffer layers, the nucleation difficulty can also be addressed by using a different growth strategy based on an off-angle substrate [78]. Such an off-angle substrate can remarkably promote the atom nucleation at the periodically distributed step edges, resulting in forming high-quality 2D materials grown directly on graphene. A good example based on this strategy is shown in the paper [78] (Figure 11). Miscut SiC substrates are used to grown graphene. Then, single-crystalline GaN films on graphene/SiC substrates are grown by using periodically distributed steps as the GaN nucleation sites. The following step is to deposit a stressor metal (Ni) and attach a thermal release tape to separate entire GaN films from the graphene surface and transfer the released GaN to host substrates. Fully functional blue light-emitting diodes (LEDs) have been demonstrated by this technique. SiC substrate reuse is also demonstrated.

Compared to the thermal, chemical, and mechanical approaches, the abovementioned strategy is a simple and feasible transfer technique with no need for additional procedures or equipment. This technique is similar to spalling, but one distinct merit is the accurate thickness control of the released layer—the thickness of the layer to be released is controlled by epitaxial growth, rather by the fracture depth decided by the stress amplitude. The other advantage is the reduced stress required for transfer, compared with spalling. Finally, the separation interface is smoother due to the 2D buffer layer not allowing covalent bonds between the epi-layer and the substrate.

#### 2.3.3. Water-Assisted Delamination

Water-assisted delamination exploits the phenomenon of water-assisted debonding at the interface between a metallic layer (e.g., Ni) and an oxide layer (SiO2) [208,209,210]. This debonding in turn lifts off the upper layer from the original SiO2/Si substrate. The underlying mechanism is due to the water-induced decrease in the critical adhesion energy of the metal–SiO2 interface, which can be over 70% [209].

**Figure 12:**
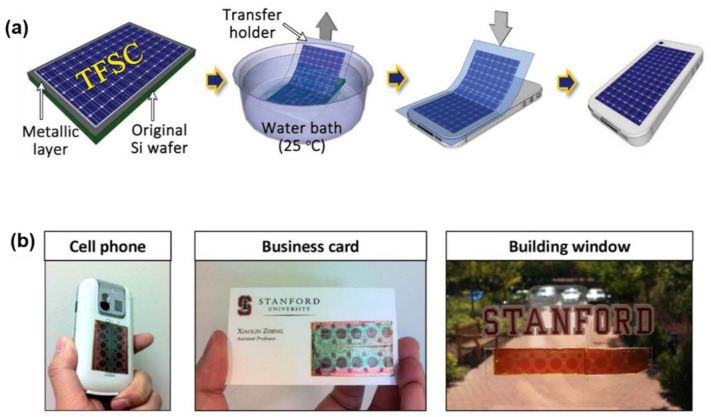
(**a**) Procedures of the peel-and-stick process. (**b**) Solar cells on cell phone (left), business card (middle), and building window (right). Adapted from [208].

The discovery of this technique can be credited to Lee and coworkers [208,209]. An example [208] of an application using this technique is the transfer of thin-film solar cells (TFSCs) onto arbitrary substrates (Figure 12). TFSC films are deposited on Ni-coated SiO2/Si substrates, followed by standard microfabrication to form TFSC devices. A tape is attached to the TFSC surface as the temporary holder. The entire sample is then loaded into a water bath. A small peeling force is then applied at the tape edge to promote water penetration into the interface, and thus inducing the delamination of the TFSC devices from the substrate. The final step is transferring the released TFSC devices to the receiver substrates by sticking and removing the tape. Based on this technique, high-efficiency solar cells transferred to arbitrary substrates, such as cell phones, business cards, and glass windows, have been demonstrated. Such transferred devices maintained the same efficiency of 7.5%, implying no obvious degradation caused by the transfer process.

**Figure 13:**
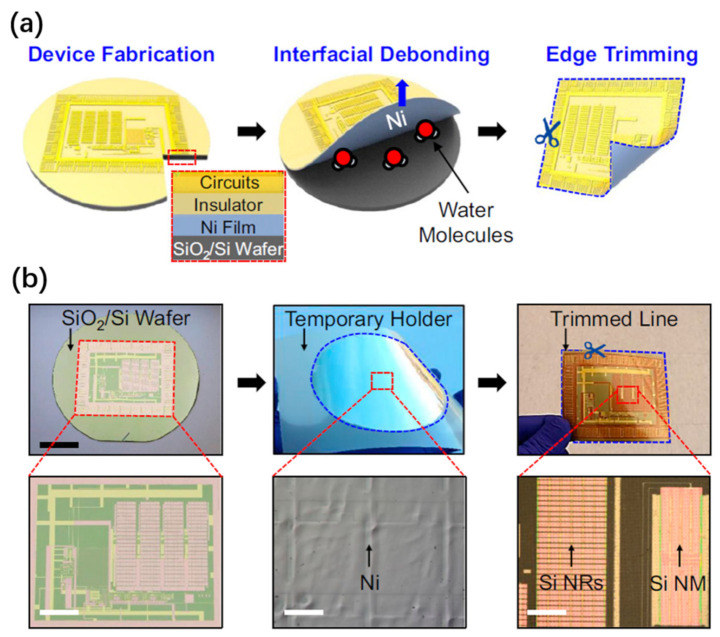
(**a**) Schematic illustrations of key steps for physically liberating thin-film nanoelectronics from a fabrication SiO2/Si wafer in water. (**b**) Optical images of the thin-film nanoelectronics on the SiO2/Si wafer (left), and peeled with a thermally releasable tape (middle), and then trimmed neatly (right). The bottom frame shows the corresponding microscope images. Si NRs in (**b**) stands for Si nanoribbons, and Si NM in (**b**) refers to Si nanomembrane. Adapted from [210].

In a more recent work [210], the same method is exploited to fabricate a wide range of thin-film nanoelectronic devices, such as a transferred Ag nanowirebased resistor, Si nanoribbon-based p-i-n diode, Si nanomembrane-based transistor, Si nanomembrane-based thin-film capacitors, nanomembrane-based MOSFETs, and a hybrid photodiode system that combines p-doped Si NM and n-doped MoS2 (Figure 13). The process has two primary steps: (i) transfer printing various single-crystalline semiconducting nanomaterials onto specific locations of a SiO2/Si wafer in a single device layout, followed by conventional CMOS fabrication to form electronic circuits on the wafer, and (ii) physically separating the entire layer of the completed thin-film nanoelectronics from the fabrication SiO2/Si wafer, which can be then pasted onto an arbitrary kind of supporting substrate or surface. The technique discussed here is wafer-recyclable, environmentally friendly, and cost-effective, showing good prospects for wafer-level production and integration of thin-film devices onto a single substrate.

### 2.4. Layer Transfer by Smart Cut

**Figure 14:**
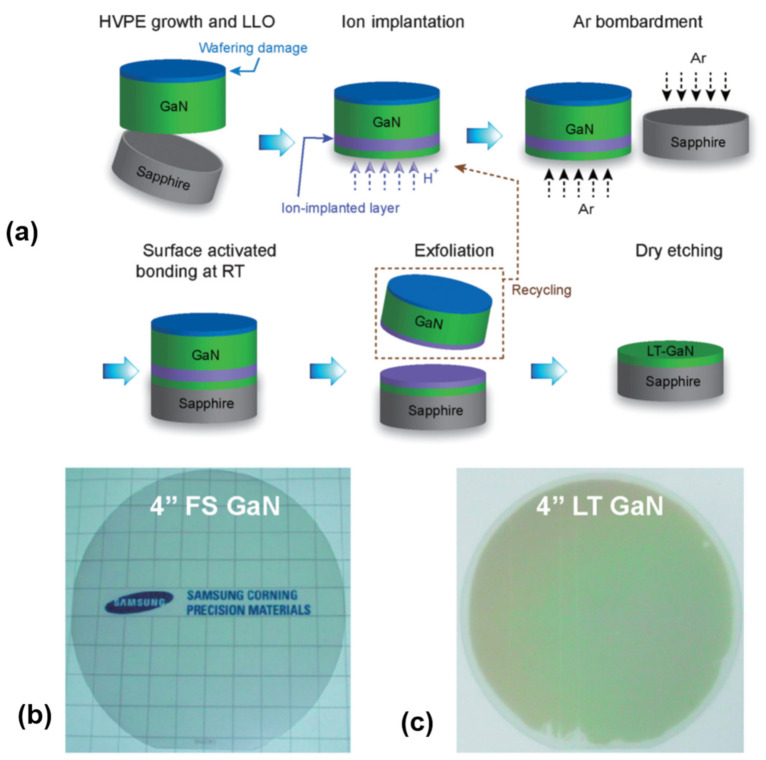
(**a**) Schematic illustrations of key steps for ion cutting techniques for GaN layer transfer. (**b**) Four-inch free-standing GaN template, and (**c**) transferred 4-inch GaN layer on the sapphire substrate. Adapted with permission from [2]. Copyright IOP Science, 2013.

Smart Cut, or ion cut, is a technique of exploiting both ion implantation and wafer bonding to transfer ultrathin single-crystal layers from a donor substrate to a receiving substrate. This technology has been commercialized for the fabrication of silicon-on-insulator (SOI) wafers for many years [87], but it has also been explored for fabricating free-standing GaN membranes recently [2,68,69,211,212,213,214,215]. Taking splitting GaN, for example, the key processing steps of ion cut [2] are schematically shown in Figure 14a. A free-standing GaN template is prepared by depositing a thick GaN layer on a sapphire substrate, followed by LLO. The N-face of free-standing GaN is then implanted with H^+^ ions after lapping and chemical mechanical polishing. Argon atom beam irradiation on the N-face GaN and sapphire surfaces under vacuum is then conducted, in order to form chemically active dangling bonds. The next step is bonding the free-standing GaN to another sapphire substrate via plasma-treated hydrophilic bonding. Afterwards, the layer transfer is carried out in a furnace by annealing GaN/sapphire. Finally, the implantation-induced damaged layer on the low temperature grown GaN (LT-GaN) surface is removed using a dry etcher, resulting in LT-GaN transferred to the receiving wafer. Based on this technique, both a free-standing GaN template and GaN layer transferred to the receiving substrate with a wafer size up to 4 inches were demonstrated, as shown in Figure 14b,c.

One of the distinguishing features of the ion cut process is the production of multiple templates from a single donor wafer, thereby reducing the cost per template without compromising the crystalline quality. Another advantage is that the layer thickness can be finely controlled with nanometer-scale accuracy. For instance, with Smart Cut techniques, wafer-scale processed silicon films in the range of 0.2 to 1 µm in thickness have been reported.

## 3. Chip-Scale Transfer Techniques

Thus far, in Section 2, we have discussed various layer transfer techniques for a particular semiconductor material of interest, which often has a large size matching the growth substrate. In many cases, chip-level devices with much smaller dimensions need to be assembled onto the receiver substrate of interest for more advanced functional systems or hybrid integration. For instance, an array of assembled micro-LEDs can be used for constructing a display module [99,118]. Lasers integrated with coupling waveguides are in high demand for developing high-performance photonic integration circuits. Furthermore, there are many cases where various chip-level components (e.g., sensors, LEDs, lasers, etc.) are required to be hetero-integrated onto the same substrate. Such complex functional systems could be achieved by single devices. There are some well-established transfer and assembly techniques for the placement of chips with a relatively large thickness and die size, such as “pick-and-place”, die bonding, and flip-chip bonding [216,217]. However, with shrinking the chip thickness and size, these methods become problematic and face serious challenging for proper chip handing, placement, and throughput. These thin chips are very fragile and tend to be easily damaged by conventional pick-and-place equipment. A few techniques have been developed to address the challenge of handing ultrathin dies, but the placement throughput is far from satisfactory. Additionally, ultrasmall chips tend to suffer from the effect of the van der Waals, surface tension, and electrostatic forces, which may be dominant compared with the external mechanical force applied by a vacuum head [122,123]. As a result, conventional mechanical placement with high accuracy and throughput becomes extremely difficult for small dies. Therefore, there is growing interest in developing feasible transfer and assembly techniques for ultrasmall and ultrathin chips.

### 3.1. Chip Transfer by ELO

**Figure 15:**
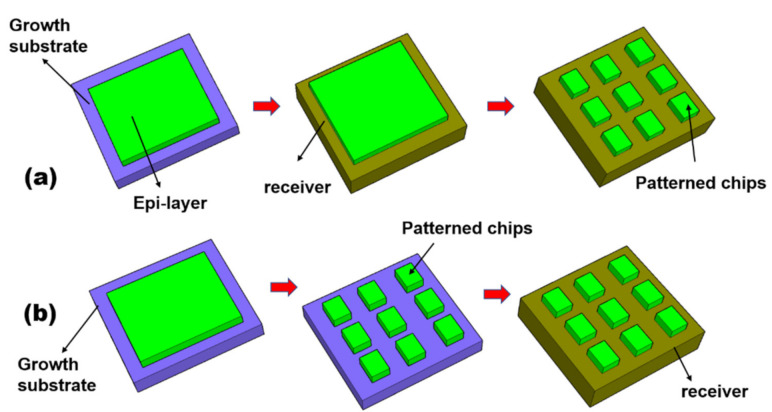
Sketches of the two methods for chip-level transfer by ELO. (**a**) “Layer-first” concept, where the epi-layer is first transferred to a receiving substrate by ELO. The transferred epi-layer is then patterned into functional chips. (**b**) “Chip-first” concept, where the epi-layer is first patterned into function chips, and then they are transferred to a receiver substrate by ELO.

The layer transfer techniques discussed earlier, with some modifications of the process, can be exploited to achieve chip-scale transfer. One straightforward approach is schematically illustrated in Figure 15a. Layer materials of interest are transferred from the growth substrate to a temporary substrate based on the aforementioned epitaxial lift-off techniques. These transferred layer materials can then be patterned and turned into discrete chips or functional devices using standard microfabrication techniques, or referring to the “layer-first” approach in the following section. In this case, the chip essentially represents a patterned layer with smaller dimensions. Further transferring of these discrete chips to the final substrate is possible by tuning the adhesion at the device/substrate interface.

An example of fabricating thin-film GaN high electron mobility transistor (HEMT) devices bonded to Si based on layer transfer technology is demonstrated by Chung et al. [20]. GaN on Si(111) is bonded to a Si(100) substrate using spin-coated HSQ photoresist as the bonding media. The growth Si(111) substrate is then removed by SF6-based plasma. This results in the transfer of a thin layer of GaN to Si(100). HEMTs are then fabricated based on the transferred GaN thin layer. These N-faced HEMTs show superior performance compared to the Ga-faced counterparts. For instance, the maximum current in the N-faced device is around 30% higher than in the Ga-faced device.

This top-down chip transfer technique, however, has several limitations. Since the discrete chips are formed after epitaxial lift-off and layer transfer, a part of the layered material is inevitably lost during patterning. An additional disadvantage is the difficulty in integrating various components. The spacing and position of each individual device cannot be flexibly adjusted since they are formed by one common masking step. Finally, scaling of the layer transfer by ELO to a large size is sometimes difficult. The primary reason is that ELO relies on lateral undercut of the release layer by wet etching, which is commonly time-consuming for wafer-scale processing due to the limited area exposed for etching. The etching can only start from the wafer edge and progressively move to the wafer center with increasing the etch duration.

To overcome the above limitations, “chip-first”-based ELO techniques have been developed, as shown in Figure 15b. In a “chip-first” ELO approach, discrete chips are fabricated on the growth substrate using standard optical lithography and microfabrication techniques. To assist the transfer, the patterned devices are commonly bonded to a temporary/final substrate using adhesive, wax, or wafer bonding. Such isolated chips are then released and transferred to the receiving/final substrate using similar ELO technologies as discussed earlier. In other words, the processing sequence is reversed compared with the “layer-first” technique. The chip-first concept has some advantages. For instance, the lift-off speed and yield can be substantially improved, compared with the layer-first concept. The primary reason is that now each chip is exposed to the etchant environment simultaneously, and thus the etching speed is accelerated significantly. A wide range of chip-scale devices, such as LEDs [1,7,8,19,29,57,63], HEMTs [10,45], and detectors [60], have been demonstrated by the ‘chip-first”-based ELO approach.

### 3.2. Chip Transfer Using Laser-Based Technologies

#### 3.2.1. Chip Transfer by LLO

**Figure 16:**
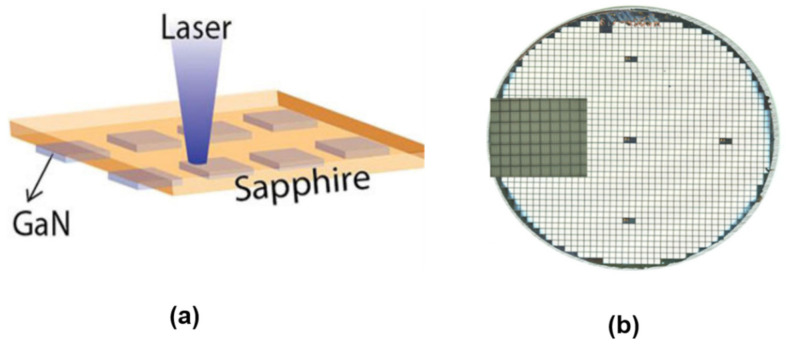
(**a**) Sketch of the LLO process for prepatterned semiconductor chips, (**b**) example of wafer-level LED chips released by LLO. Adapted with permission from [218]. Copyright IOP Publishing, 2009.

Similar to the layer transfer by laser lift-off, LLO is also applicable for chip-scale transfer. The major difference is the need to form discrete chips on the growth substrate prior to LLO (Figure 16a). Attaching the wafer with such prefabricated chips to a temporary/permanent support, followed by laser scanning from the backside of the substrate, allows the chips to be released from the substrate and transferred to the temporary/permanent substrate. The “chip-first “concept has some advantages over the “layer-first” approach. First of all, releasing isolated small chips by LLO is much easier than a large layer, due to the stress release when patterning the isolated chips. Second, the LLO yield of chip-level processing is generally much higher. Even if there are localized defects or non-functional devices after laser lift-off, these non-functional chips are easy to remove.

One representative application of this technique is the fabrication of high-power thin-film flip-chip (TFFC) LEDs. Lee et al. demonstrated vertical high-power LEDs transferred to a conductive, permanent metal support by LLO [218] (Figure 16b). Chip-scale high-brightness LEDs are formed on sapphire substrate. They are then bonded using a bonding metal alloy consisting of Au, Sn, and Cu to a silicon wafer topped with a layer of titanium, followed by LLO to remove the sapphire substrate. The crucial metal bonding layer not only helps to assist the LLO, but also acts as a good heat sink to improve the heat dissipation. Such TFFC LEDs have been further developed by the chip maker Philips Lumileds [83], which are now commercially available in large-volume production for the lighting market.

By shrinking the LED chip size, the LLO technique can be exploited to fabricate ultrathin micro-LED chips, with the major purpose of developing high-resolution micro-LED displays [98,118,130,219,220]. For instance, Kim et al. developed a protocol to transfer predefined GaN micro-LEDs to Si by a hybrid approach of combining wafer bonding, LLO, and transfer printing [130]. In this work, Pd-In was used to bond the device to a Si carrier. Laser lift-off was done to remove the sapphire substrate. The next step was to undercut the isolated LED chips by wet etching of the bonding layer underneath. Finally, these tethered chips were picked up using a PDMS stamp and transferred to the final substrate to build functional systems. Alternatively, wafer bonding and LLO are used to transfer lateral LEDs to a temporary substrate, and then transferred to the final substrate by debonding the temporary support [98,219]. While conceptually feasible, these methods are very complicated, and involve the use of expensive wafer bonding, debonding, and transfer printing tools, which are not always available to regular users.

**Figure 17:**
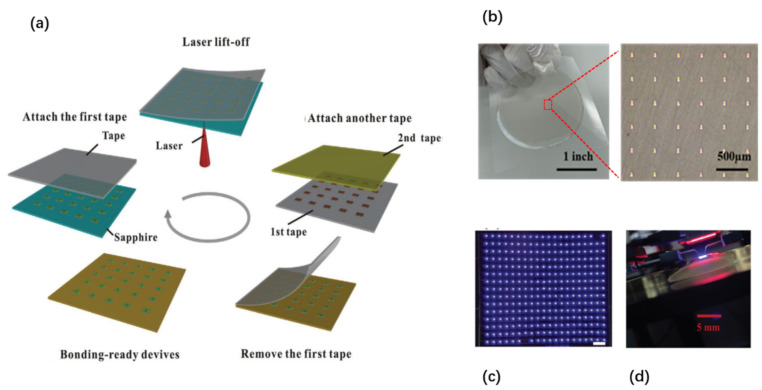
(**a**) Working principle of the tape-assisted laser transfer, (**b**) images of wafer-level micro-LED chips via tape-assisted laser transfer (TALT), (**c**) a planar display device, and (**d**) a flexible display device developed based on TALT. Adapted with permission from [118]. Copyright John Wiley and Sons, 2020.

More recently, Pan et al. [118] developed a different approach based on tape-assisted laser transfer (TALT) to address this challenge (Figure 17a). The prepatterned devices with the substrate are bonded to a temporary adhesive tape, followed by laser lift-off. The devices on the temporary tape are further flipped over to another tape, which has larger adhesion strength. Removing the first adhesive tape results in the transfer of the devices to the second tape. Such devices are highly compatible with the subsequent bonding, since the two electrodes are facing outwards. The TALT technique has eliminated the complicated and expensive wafer bonding and debonding process as required in the common processes mentioned above. It only involves in the use of low-cost tapes as the support for LLO, which can be taken off by simple peeling. Therefore, this technique can significantly simplify the transfer process and reduce the cost. Indeed, wafer-scale micro-LED transfer capability has been demonstrated (Figure 17b), showing the potential for large-volume production. Furthermore, both rigid displays and flexible displays have been demonstrated (Figure 17c,d). This technique represents a remarkable improvement over other micro-LED mass transfer techniques.

One notable feature of LLO is the ability for selective transfer [105,118,219,220] by controlling the beam scanning pattern, which is particularly useful for adjusting the spacing of transferred objects. The beam patterns can be either controlled by sequentially moving the laser beam [105,219,220], or using a shadow mask to block unwanted laser beams [118]. The latter strategy has improved transfer speed, since mechanical moving of the laser beam is a time-consuming process [118].

Apart from the miniaturized micro-LEDs, a wide range of chip-scale devices transferred to flexible substrates can also be demonstrated by LLO techniques [124,182,221,222,223,224,225]. These devices may find applications in a wide range of areas such as foldable displays, wearables, and electronic skins.

#### 3.2.2. Chip Transfer by Laser-Induced Forward Transfer (LIFT)

**Figure 18:**
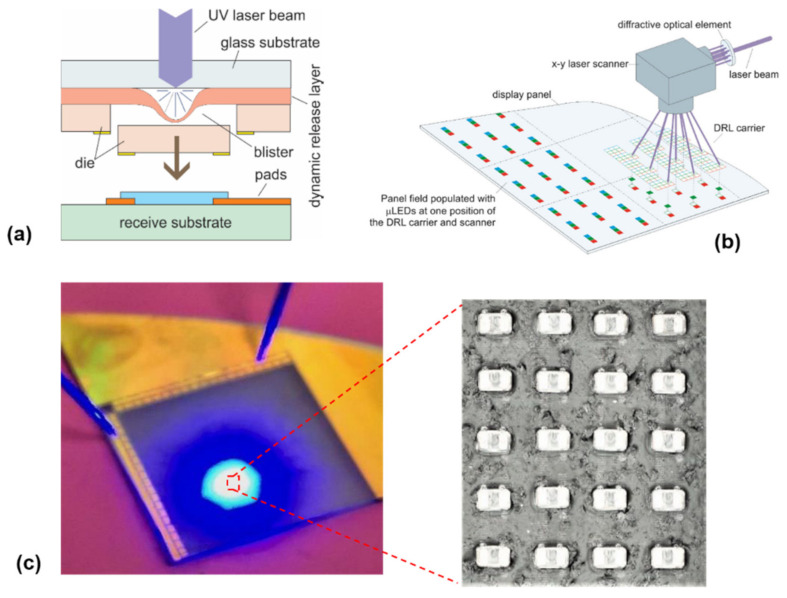
(**a**) Working principle of the laser-induced forward transfer (LIFT), (**b**) a schematic illustrating the LIFT based on using a multiple beam scanning strategy. (**c**) transferred and bonded 55 × 32 × 6 µm µLEDs on a test substrate by LIFT. Adapted with permissions from [229]. Copyright John Wiley and Sons, 2018.

Thus far, the above techniques have been based on laser ablation and decomposition of the absorption layers inside the functional chip to assist the chip transfer. There is a different laser transfer mechanism which can be exploited for chip assembly, that is, laser-induced forward transfer (LIFT) [226,227,228,229]. The LIFT starts by depositing a dynamic release layer (DRL) on a laser-transparent substrate. The chips to be transferred are then fixed on the DRL layer (Figure 18a). Ablating a small portion of the DRL layer from the substrate side using a pulsed laser results in forming a blister in the DRL, which in turn generates gas byproducts. The gas generated in the localized region serves as a mechanical actuator to push the chip toward the receiving substrate placed in close proximity.

The LIFT technique, compared with other transfer techniques, has some advantages, including fast transfer speed and relatively small placement error. The transfer speed of more than 100 M units/h and a placement error of 1.8 µm have been achieved by LIFT [229]. Furthermore, this method allows the selective transfer of every chip by manipulating the laser scanning pattern. The transfer speed can be further improved by using a multiple beam scanning strategy, which, for example, can be achieved by splitting a single laser beam into an array of laser beams using diffractive optics, as schematically shown in Figure 18b. Additional advantages of this technique include in situ bonding and defect repair capabilities. These distinct merits imply that the LIFT technique is promising for the assembly of large panels where a large quantity of devices and high assembly speed are required, for instance, for a micro-LED 8K TV. An example of the successful assembly of 55 × 32 µLEDs onto the receiving substrate by this technique is shown in Figure 18c. One notable disadvantage of this technique, however, is the laser-induced residuals appearing on the surface. Such residuals may cause contamination and affect the postprocessing procedures if required.

### 3.3. Chip Transfer by Stamp Transfer Printing

Stamp transfer printing is another technique which has been extensively explored to assist chip-level transfer [103,161,230,231,232,233,234,235,236,237,238,239,240,241,242,243,244]. This technique exploits the use of an elastic stamp to pick and place tethered devices, also known as microtransfer printing (µTP), which was invented by Prof. John Rogers. This technique has witnessed great success in assembling a wide range of materials and devices onto the targeting substrate and creating diverse hetero-integrated multicomponent functional systems which are difficult to be realized by any other assembly technologies. Existing reviews mainly focused on the stamp transfer mechanism, materials, and applications [106,139,140,161,239,245]. Here, we focus on reviewing the important device structures and stamp structures that are essential for high-yield and high-accuracy placement of the ultrathin and ultrasmall dies.

#### 3.3.1. Stamp Transfer Printing Principles

**Figure 19:**
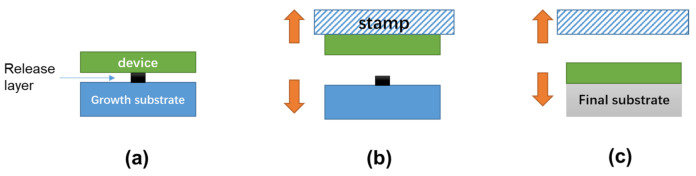
Working principle of the stamp transfer printing, including 3 major steps: (**a**) forming printable inks, (**b**) picking up the ink by a stamp, and (**c**) releasing the ink onto the receiving substrate by retrieving the stamp slowly.

Chip transfer by a stamp is enabled by three primary processes [230], as schematically shown in Figure 19. First of all, releasable device chips with tethers [243,246], or inks, are commonly formed by chemically or photochemically etching the sacrificial layer. Second, such tethered chips are picked up by mechanically breaking the tethers using various stamps. Finally, chip transfer is achieved by moving the stamp down with the devices to contact the receiver substrate, followed by lifting up the stamp slowly.

The stamp can exhibit increased adhesion by accelerating the pulling speed of the stamp, and therefore can retrieve the tethered devices from the donor substrate [230]. On the other hand, slowly moving the stamp down can weaken the adhesion of the device/stamp interface, which therefore allows the transfer of the devices to the receiving substrate. In other words, kinetically modulating the interfacial adhesion difference between the stamp/device interface and the device/substrate interface is the key factor determining whether the device (or ink) can be picked up or printed [230,247]. To assist the transfer, the final substrate is most commonly coated with an adhesive layer, for which the adhesion strength is stronger than the PDMS stamp [248,249,250]. In some cases, successful transfer to the final substrate even without using this adhesive layer is also reported, based on a VDW bonding mechanism [103,233]. The latter method is less reliable in terms of the transfer yield since the VDW bonding is highly dependent on the roughness and morphology of the device/substrate interface.

#### 3.3.2. Releasable Chip Structures

**Figure 20:**
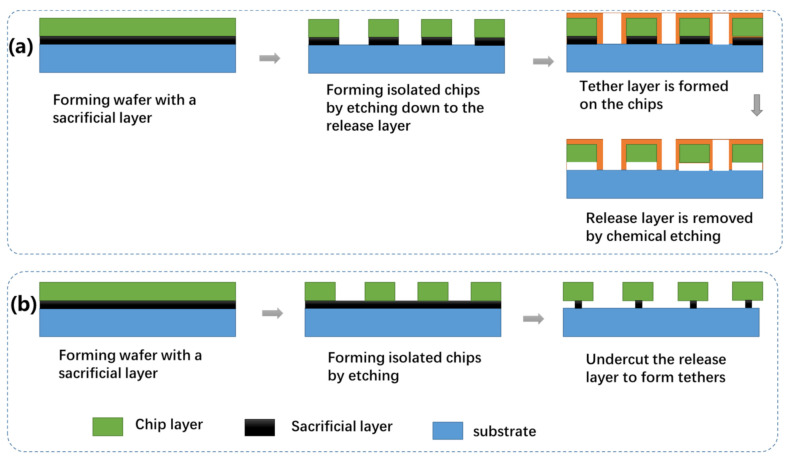
Two methods to form releasable inks with tether structures, where the tether material is introduced externally (**a**), and the tether material is from the epi-layer stack (**b**).

As shown above, forming releasable chips is one of the key processes to be developed. Among various methods, the ELO techniques discussed earlier can be adapted to fabricate tethered chips by etching the release layer which is intentionally introduced prior to growing the semiconductor layer [243,246]. Two approaches can be used to form tether microstructures, as schematically shown in Figure 20. The tethers, which hold the suspended chips in place, are formed in such a way that the release layer is selectively removed and the isolated chips are weakly bonded to the original growth substrate by the tether structures introduced externally (Figure 20a). Various material can be used for the tethers, including photoresistant, polyimide, and dielectric layers [99,250]. Sometimes, the sacrificial layer itself can also be used for the tether structure (Figure 20b). In the latter case, the sacrificial layer is laterally undercut such that only a small part of the sacrificial layer is kept to hold the suspended chips in the original position. Applying an external force by using a stamp will fracture the anchored release layer, thereby making the devices transferrable.

While suspended chips held by tethers are one of the common releasable chip structures, sometimes the releasable chips are made in a manner without tethers, and are only weakly bonded onto a temporary support by adhesion (more often it is an adhesive tape). For instance, the releasable chips can be transferred to a tape by LLO [124,223,224], using a process similar to the TALT technique [118]. These weakly bonded devices can then be picked up by stamps.

#### 3.3.3. Stamp Materials, Structures, and Fabrication

The stamp must have controllable adhesion capability for chip transfers. Being a switchable dry adhesive which can be reused for many cycles, PDMS is one of the stamp materials used most frequently [230]. While huge success has been achieved, the PDMS stamp also has some constraints. Since the adhesion is induced by the pulling speed, accurate control of the adhesion amplitude is difficult. The additional disadvantage is the deformation of the elastic stamp when it is subjected to external forces, which in turn causes the displacement of the transferred devices. For these reasons, a wide range of other stamps made from different materials have also been explored [102,135,242,251,252,253,254,255,256], including polyimide, gecko-inspired adhesive, tape, polymethyl methacrylate (PMMA), shape-memory polymer, etc. Some of them, such as gecko-inspired adhesive and shape-memory polymer stamps, have reversible adhesion switching capabilities, which means they can be used for repeated transfer processes [135,253,257]. Such capabilities are essential for the sequential assembly of devices on a large scale. Other stamps, for instance, PMMA and tapes [242,252], are commonly used for only in single printing processes. The primary reason is that the surface adhesion, once used, is changed and not fully recoverable in most cases. An additional disadvantage is the potential chance of introducing particles and contamination to both devices and receivers. Therefore, while small-scale transfers have been demonstrated due to the large adhesion strength of such stamps, they are not realistic for sequential or repeated printing required for large-area assembly. By contrast, the PDMS stamp, if the surface is well cleaned and well preserved, can be reused for many cycles.

**Figure 21:**
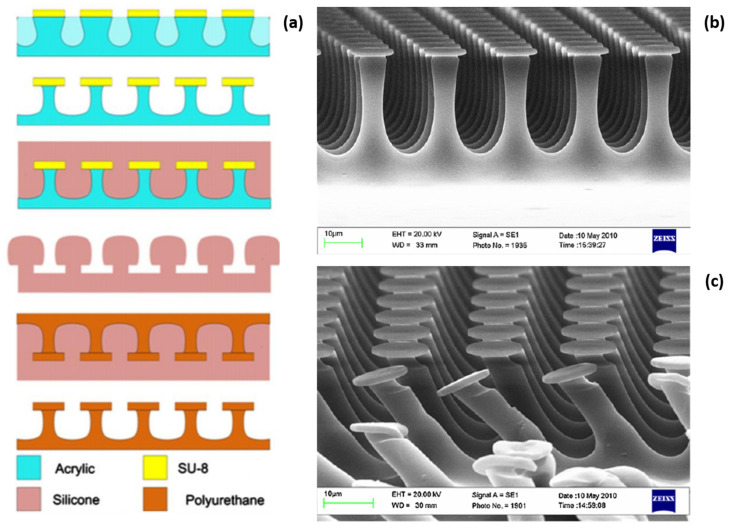
(**a**) A general fabrication procedure for making mushroom-like adhesive stamps. (**b**) SEM image of the resulting mushroom-like microstructures, and (**c**) SEM image of the stamp surface with tilted mushroom-like pillars formed by angled exposure. Adapted with permission from [256]. Copyright IEEE, 2012.

The stamp is commonly flat, which is suited for printing a block of chips within the stamp area. In some cases, however, the stamps are patterned with microstructures to enhance the adhesion switchability [258]. For example, a stamp with extruded pyramid-shaped microstructures was developed to enhance the adhesion switchability [233]. Kim et al. reported a mushroom-like stamp [258]. In another study, an inflatable stamp array with active pressure control was developed [259]. In all cases, the adhesion is modulated by changing the contact area of the microstructure with the device upon external pressure. Such stamps with patterned microstructures are commonly formed by molding, combined with conventional lithography techniques [233,256,258,260]. For example, a stamp with micropyramids is formed by pouring PDMS precursor into a wet etching-defined pyramid-like silicon template [233]. Due to the anisotropic wet etched nature of the silicon under KOH solvent, pyramid-like apertures can be formed in the silicon surface using a hard mask. Molding liquid polymers such as PDMS against this patterned silicon template, followed by curing and demolding, results in the formation of the required dry adhesive stamps. Figure 21a illustrates an example of the fabrication procedure for forming mushroom-like adhesive structures by undercutting the acrylic layer below the SU8 photoresist [260]. The resulting mushroom pillars are shown in Figure 21b. The same molding technology, combined with angled exposure, can be further extended to form stamps with tilted pillars (Figure 21c). The adhesion of such a stamp with tilted pillars can be notably increased by shearing in one direction, but it becomes substantially weakened in the opposite direction [258,260]. While these multiscale fiber structures show improved adhesion performance, they are generally more difficult to be scaled up to a large stamp area, and also add fabrication complexity.

#### 3.3.4. Variants of the Stamp Transfer Printing Techniques

Modifications of the stamp transfer printing technique have also led to the evolution of a wide range of other chip transfer approaches, including tape-assisted transfer printing [102,242,251], roll-to-roll printing [100], and laser-driven non-contact printing [261]. Most of these methods rely on the modulation of the interfacial adhesion strength of the stamp/device interface.

Tape-assisted transfer exploits the use of commercial tapes to replace conventional PDMS stamps for chip-scale transfer [102,144,242,251]. Such tapes commonly have larger adhesion switchability than conventional PDMS stamps, and therefore they are well suited for high-yield pickup of the devices. Device releasing, on the other hand, can be achieved by weakening the adhesion of the tape. Depending on the specific tape, the weakening of the tape’s adhesion can be achieved via different methods, such as temperature control, UV illumination, and solvent soaking. For example, Yan et al. demonstrate the use of a thermal release tape (TRT) for device transfer [102], for which the device release is achieved by heating the receiving substrate. The adhesion of the TRT can be substantially weakened by raising the substrate temperature. In another example, the use of water-dissolvable tape for chip-scale transfer was demonstrated [242]. In this case, the adhesion can be reduced by simple water soaking. Chip-scale devices are therefore able to be transferred to the receiving substrate. Compared with PDMS stamp transfer, the tape is generally commercially available at a much lower cost and does not involve the complicated stamp fabrication needed for the PDMS stamp. Therefore, tape-assisted transfer is easier to implement. However, the adhesion of these tapes, once lost, is not recoverable in most cases. This means they are more suited for one-time transfer instead of repeatable transfer. In some cases, the transfer accuracy is unsatisfactory, which may cause registration issues. The transfer speed is also compromised since the adhesion weakening takes a longer time than other approaches.

**Figure 22:**
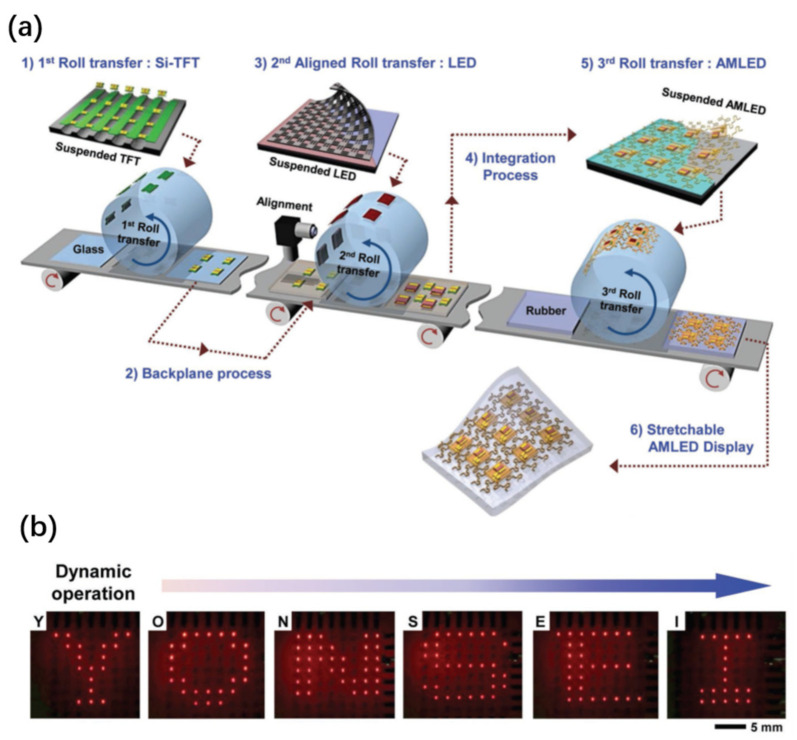
(**a**) A general roll-to-roll transfer printing process for making flexible active matrix LED (AMLED) devices integrated onto the thin-film-transistors (TFTs). (**b**) Emission images of the flexible micro-LED arrays assembled by roll-to-roll printing. Adapted with permission from [100]. Copyright John Wiley and Sons, 2017.

Roll-to-roll printing is another modified printing technique which is based on a cylinder stamp, instead of the planar stamp used in the conventional transfer printing [100]. Compared to the planar stamp printing, a cylinder stamp in a roll-to-roll printing system has the merits of larger area scalability and higher printing productivity. An additional benefit of this technique is the precise control of the contact area and contact uniformity through a feedback module, which is essential for improving the printing yield and placement accuracy. An example of using this technique for developing flexible microscale LED displays is demonstrated by Choi et al. [100]. The process has three successive printing steps: (i) printing Si-thin-film-transistors (TFTs) onto a carrier substrate, (ii) printing LEDs onto the same carrier substrate, and (iii) forming a display by interconnection of the TFT and LED, followed by transfer printing of the display from the carrier substrate to the final rubbery substrate (Figure 22a). This enables the fabrication of high-performance stretchable LED displays (Figure 22b).

**Figure 23:**
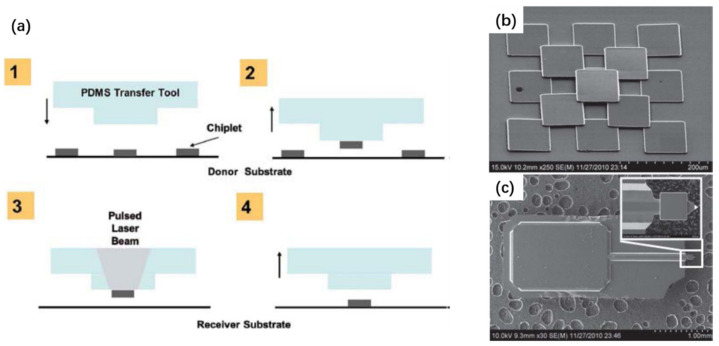
(**a**) Working principle of the laser-driven non-contact transfer printing based on a polydimethylsiloxane (PDMS) transfer head. (**b**) Three-dimensional pyramid built with silicon squares. (**c**) Silicon square placed on a silicon cantilever. Adapted with permission from [261]. Copyright IEEE, 2012.

Thus far, the above chip transfer techniques rely on the direct contact of the stamp with the device. The contact-based techniques, however, have some disadvantages. For instance, multiple contacts may cause contamination to both the stamp and the device, which in turn form defects and cause the failure of the subsequent device integration. Transfer printing operated in contact mode is difficult to apply to curved substrates. Furthermore, repeated contacts can cause remarkable stress to the device, which in turns breaks the thin chip. For these reasons, non-contact printing techniques have also been explored [261]. An example of the printing techniques operated in a non-contact mode is laser-driven non-contact printing developed by Saeidpourazar et al. [261] (Figure 23a). Chiplets are picked up by a PDMS stamp. A pulsed laser beam is irradiating from the stamp top. The laser beam is transparent to the stamp whereas it is absorptive to the device. Due to the different thermal responses between the PDMS stamp and the chip, the chip can be delaminated and eventually released from the stamp to the receiver substrate. As the pulsed laser does not cause a notable rise in the temperature in the device, the potential damage to the functional chips can be minimized. With this technique, complex 2D or 3D assembly of chips can be demonstrated (Figure 23b,c).

#### 3.3.5. Applications of the Stamp-Transfer Techniques

**Figure 24:**
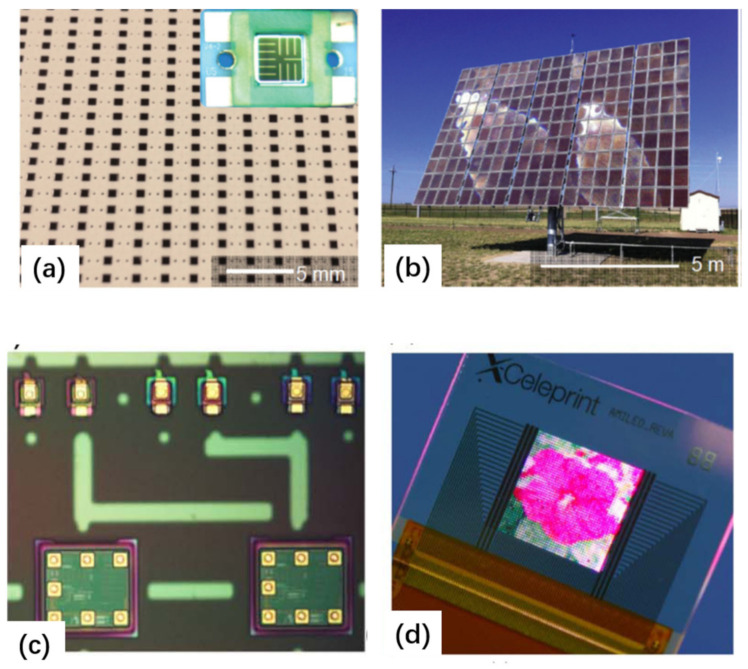
(**a**) Photograph of transfer-printed multijunction GaAs solar cells on a low-cost ceramic sub-mount substrate. The inset shows a magnified view. (**b**) A pilot-scale commercial concentrator module with a certified efficiency of 35.5%. Adapted with permission from [106]. Copyright John Wiley and Sons, 2015. (**c**) A transfer-printed unit consisting of RGB micro-LED subpixels and corresponding driver IC. Adapted from [99] (**d**) Active micro-LED display made from the printed units shown in (**c**). The interconnection is based on electroplated copper. Adapted from [99].

Stamp transfer printing is a versatile chip-scale assembly technique which enables heterogeneous integration of a wide range of optoelectronic devices, ranging from LEDs, lasers, solar cells, and detectors, to complex functional systems [106,139]. This technique can overcome the limits in conventional assembly techniques and create many new functionalities and boost the system performances of existing ones. Solar cell panels [106] and emissive display screens [99] are two examples of such complex systems with added functionality and enhanced performance. The transfer-printed multijunction microscale solar cells shown in Figure 24a have been exploited for assembling a pilot-scale commercial concentrator module, which has a recorded efficiency up to 35.5% (Figure 24b). Figure 24c,d shows an active-matrix display consisting of discrete micro-LEDs, which are assembled by stamp transfer printing, followed by interconnection of these micro-LEDs via metal wiring. The small micro-LEDs produce sufficient amounts of light for high luminance while occupying a small fraction of space in the display, therefore allowing the panel to be integrated with miniaturized integrated circuits for drivers and other device components for extra functionality.

**Figure 25:**
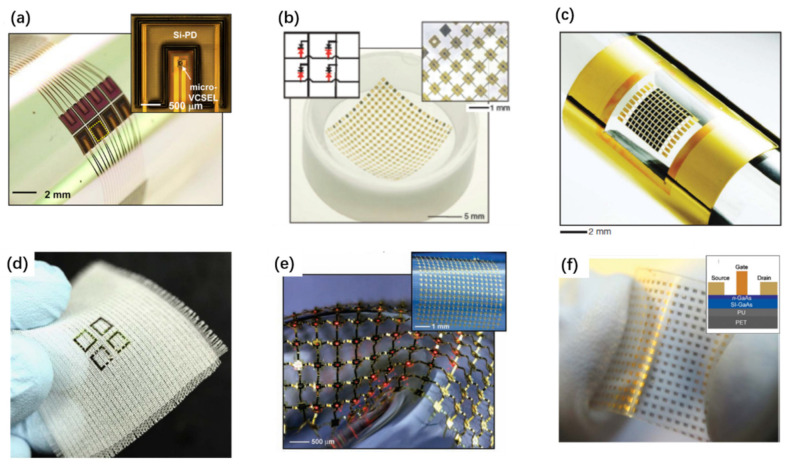
(**a**) A fluorescence sensor on PET wrapped around a cylindrical support interconnected by Cr (15 nm)/Ag (1500 nm)/Au (30 nm). Adapted with permission from [175]. Copyright American Chemical Society, 2016. (**b**) A silicon photodiode array integrated on a hemispherical glass substrate as an electronic camera eye, which is interconnected by chromium–gold–chromium. Adapted with permission from [264]. Copyright Springer Nature, 2008. (**c**) A solar module consisting of a 10 × 10 array of GaAs solar cells on a PET substrate. Interconnected by 30nm Cr/350nm Au. Adapted with permission from [262]. Copyright Springer Nature, 2010. (**d**) A stretchable indium gallium zinc oxide-based electronic textile formed by cilia-assisted transfer printing, interconnected by Cr/Au. Adapted from [263]. (**e**) Flexible AlGaInP micro-LED arrays interconnected by Ti (20 nm)/Au (300 nm). Adapted with permission from [250]. Copyright The American Association for the Advancement of Science, 2009. (**f**) Flexible GaAs wire-based metal–semiconductor field-effect transistor (MESFET) printed on a plastic substrate. Adapted with permission from [265]. Copyright AIP Publishing, 2005.

Not only is hetero-integration on a planar substrate possible, this stamp transfer technique is also a powerful technique for integration onto curved surfaces [129,175,250,262,263,264,265]. As a consequence, a wide range of flexible optoelectronic devices can be produced (Figure 25). For example, flexible optofluidic fluorescence sensors (Figure 25a) consisting of GaAs-based vertical-external-cavity surface-emitting-lasers (VECSELs) and silicon photodiodes have been integrated onto a plastic polyethylene terephthalate (PET) substrate, illustrating the capability of this technique [175]. Such hetero-integrated devices show minimized performance degradation without compromising intrinsic materials properties. In another example, photodetector arrays printed onto hemispherically curved surfaces (Figure 25b) have been demonstrated [264]. Such devices, with configurations similar to those of mammalian eyes, have attracted intensive interest in digital imaging, thanks to the ability of this geometry to match Petzval image surfaces associated with simple lenses. Such devices would be difficult to achieve by conventional technologies since most of the standard growth, deposition, and fabrication processes are established on planar surfaces of substrates. Yoon et al. [262] demonstrated GaAs-based flexible solar cell arrays integrated onto a PET substrate by transfer printing, combined with ELO (Figure 25c). The cells can be assembled in series and/or parallel configurations to produce output power at high or low voltages, implying an important advantage of the use of small cells arranged in large array format. It is also possible to fabricate electronic textiles based on transfer printing [263] (Figure 25d). Artificial cilia are introduced in this work as adhesive elements to facilitate the fabrication and integration of electronic devices onto the woven fabric by transfer printing. Such devices can fit the human body comfortably, and deform naturally upon movement, showing good prospects for developing wearable devices. Park et al. [250] developed flexible interconnected AlGaInP micro-LED arrays by transfer printing, where the interconnections are supported by arc-shaped bridge structures that can deform in response to applied strain (Figure 25e). Such flexible LED devices open up the prospect of foldable displays and biomedical applications. Sun et al. [265] demonstrated the transfer printing of GaAs micro/nanowires for bendable metal–semiconductor field-effect transistors (MESFETs) on plastic substrates (Figure 25f). Such devices may find a wide range of applications, including displays, sensors, medical devices, etc.

**Figure 26:**
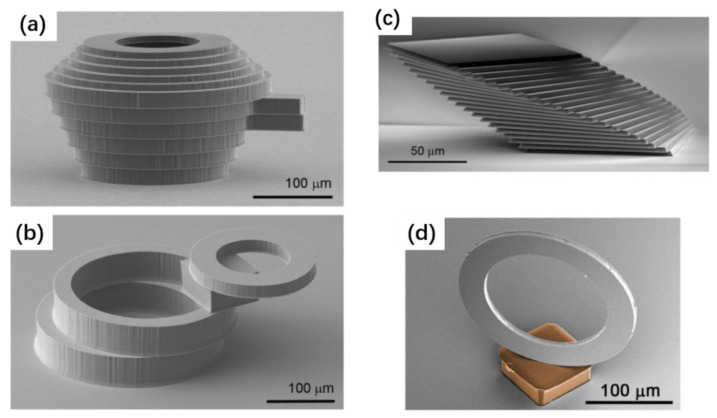
Examples of complex 3D objects formed by transfer printing. (**a**) Stacked silicon rings with varied thicknesses and diameters. Adapted from [266]. (**b**) A combination of silicon rings and a silicon square block. Adapted from [266]. (**c**) Multilayer configurations of 3 µm thick silicon platelets in a single stack with small incremental rotations and translations. Adapted from [233]. (**d**) A vertically aligned Si ring joined to an SU-8 resist block. Adapted with permission from [267]. Copyright IOP Publishing, 2012.

The stamp transfer technique also allows the creation of 3D objects [233,261,266,267]. Various complex 3D objects formed by multiple printing, stacking, and joining of microscale parts or thin films made from either the same materials or dissimilar materials are shown below (Figure 26). In several studies, 3D electronics have also been achieved by stamp transfer printing [224,248]. These results indicate that stamp transfer printing has advantages such as extraordinary placement accuracy and capability for forming complex configurations, opening up a wide range of potential applications in 3D integration and packaging.

### 3.4. Fluid-Assisted Chip Transfer

**Figure 27:**
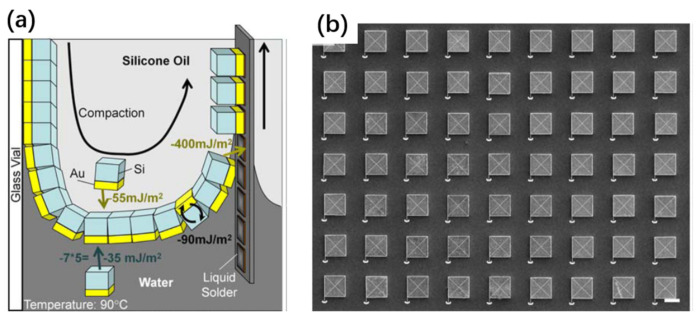
(**a**) Mechanism of surface tension-directed self-assembly at a liquid–liquid–solid interface. (**b**) SEM image of Si chiplets assembled in regular arrays. Adapted from [276].

Surface tension of a fluid can be exploited to direct the self-assembly of small components at predetermined locations [95,96,97,268,269,270,271,272,273,274,275,276,277,278,279]. An example of surface tension-directed fluidic assembly of microscale parts in predefined regions is shown below [276,277] (Figure 27). The assembly is achieved by pulling the sample upward through the liquid–liquid–solid interface. The process uses a stepwise reduction of the interfacial energy to transport the chips from a suspension to the interface, preorient the chips in the right direction, and assemble the chips on a substrate with patterned solder [276]. As a result, high speed (62,500 chips/45 s), high accuracy (0.9 micrometers, 0.14°), and high yield (>98%) of the assembly of very small parts of sizes of only 20 µm can be achieved.

Several variants of the above fluidic assembly technique have also been developed. These include: shape-directed fluidic methods based on predesigned locations [272], liquid solder-based self-assembly [273], capillary force-directed self-assembly based on hydrophilic/hydrophobic surface patterns [268], and/or their combinations [269,270]. These methods can be extended to achieve hetero-integration of components with different sizes [272,277]. Furthermore, a wide range of devices can be constructed by fluid assembly onto either planar surfaces or curved surfaces, including LEDs [269,270,271,274] and solar cells [276,277]. Complex functional systems can also be demonstrated by this technique, such as curved displays [280] and rubber-like LED lighting modules [97], highlighting the versatile capability of this technology in 3D assembly.

Being a self-assembly process in nature, fluidic assembly is very fast, and the assembly can be finished within minutes. This method also has excellent scalability in assembly areas, and can overcome the limitation that conventional chip transfer techniques work poorly on curvy surfaces. However, when the sample is thin and small, the surface tension may dominate over its weight, making the assembly difficult. An additional disadvantage is the difficulty in selective transfer and defective chip repair. For these reasons, the fluid-assisted assembly techniques are commonly used for assembling components with relatively large sizes (over 100 µm) and large thicknesses (over a few hundred microns).

### 3.5. Electrostatic-Assisted Chip Transfer

Electrostatic assembly exploits the adhesive force induced by an external electric field across a set of conductive electrodes to manipulate microcomponents [281,282,283,284,285,286,287,288,289]. In other words, charged microcomponents can be trapped by patterned surface areas with localized electrical fields. Electrostatic force has been known for years. Several investigations into electrostatic field force have mainly been focused on manipulation of microparticles [284]. However, this concept was also explored for macroscale wafer handling [283] and microscale device assembly [281,283,286,287,288,289].

**Figure 28:**
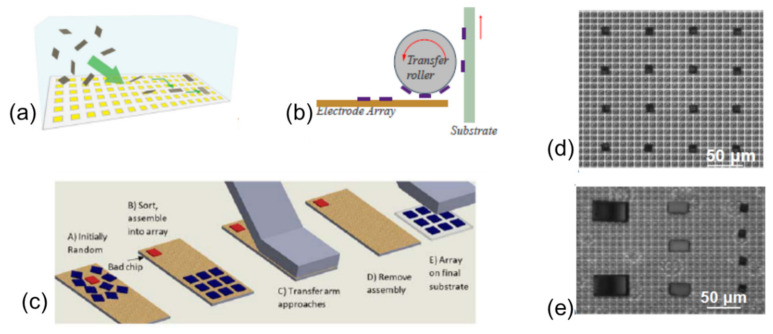
Schematic view of the microassembly system based on electrostatic field, where micro-objects (**a**) initially in solution are manipulated with electrode arrays (yellow) and then (**b**) transferred to a final substrate with a roller or continuous belt process. (**c**) Flat contact stamp transfer. (**d**) Chiplets assembled in an array and (**e**) heterogenous chiplet assembly of chips of different sizes. Adapted with permission from [289]. Copyright IEEE, 2019.

Recently, the commercial feasibility of this technique for microchip manipulation has been investigated. For instance, PARC has developed a microcomponent printer based on this concept [289] (Figure 28a–c). Microscale chips are suspended in aqueous solvents. A phototransistor array is designed to create an addressable electric field, which can manipulate the charged chips in parallel with the help of agitation. The assembled microchips can then be transferred to the target substrate using either a flat rubber stamp or a roller belt-based non-contact electrostatic system. As an example, silicon chiplets with either identical dimensions or of different sizes down to 10 µm can be accurately assembled onto the target substrate (Figure 28d,e).

Compared with other assembly techniques, the static electric technique can rapidly sort, place, and orient micro-objects of extremely small sizes into custom patterns. The technique has potentially the benefit of high throughput and low cost as it is a truly programmable assembly technique, which allows fast manipulation of small chips in parallel. However, accurate manipulation of the electrostatic force is difficult. There is evidence that excessive electrostatic force may damage the chips, leading to the failure of the functional device.

## 4. Summary and Outlook

We have summarized a variety of hetero-integration technologies of semiconductor materials and devices by layer transfer and chip transfer. Among them, some layer transfer techniques, such as epitaxial lift-off and stress-induced delamination, allow the practical wafer-scale transfer of ultrathin semiconductor films in a cost-effective manner while preserving the original growth substrate, making them appealing for practical volume production. Other chip-level transfer methods provide practical ways to enable the hybrid assembly of dissimilar materials and components to build complex functional devices and systems, regardless of the conditions for their growth and fabrication. In many cases, such functional systems, which are difficult to achieve by conventional assembly technologies, show improved device performances and expanded functionalities. For instance, some of the methods presented in this review, such as stamp transfer printing, allow the conformal integration of microscale components onto flexible substrates and curved surfaces, opening up the possibility of demonstrating complex microdisplay systems and flexible optoelectronic devices. Fluid-assisted assembly, on the other hand, allows the accurate assembly of components at predesigned locations by exploiting liquid surface tension force or liquid solders, and thus generates programmable device patterns and systems as desired.

Despite of the great progress in layer and chip scale transfer techniques, there are many challenges to be overcome for these methods, such as issues relevant to transfer speed, batch processing, transfer accuracy, transfer yield, and assembly cost. Since each transfer method has its own advantages and limitations, care must be taken to choose the right layer or chip transfer method for a specific application. Additionally, the optimization of the existing methods and development of new methods would motivate continued and expanded efforts in the future. The following research directions are worth exploring further:

Large-scale layer transfer techniques. Several techniques have exhibited capability for thin-film transfer, but the transfer is limited to only very small areas, restricting their practical use in large-scale production. Furthermore, the degraded thin-film quality, often accompanied by increased roughness and deteriorated optical performance, is another critical factor limiting these methods for practical use. ELO represents a promising technique for wafer-scale layer transfer technology. Continued efforts, however, should be focused on exploring the advanced epitaxial techniques by introducing suitable sacrificial layers to assist fast ELO while ensuring that the quality of the epitaxial layer is not obviously degraded. The corresponding etchants should be also properly selected, such that the etching duration and chemical etching-induced damage are minimized. VDW-assisted epitaxy is another promising method to be explored further for wafer-level layer transfer. However, the epitaxy on 2D layered semiconductors remains challenging. Therefore, continued efforts should be dedicated to choosing the right 2D sacrificial layer and improving the VDW epitaxy growth dynamics by optimizing the growth parameters and minimizing the dislocations at the interface.

Chip-scale transfer with high yield, high accuracy, and high throughput. Chip-level transfer techniques are critical for hetero-integration of various components when creating multifunctional devices and systems. Existing challenges, however, are mainly relevant to the low transfer yield and throughput. Efforts will be therefore focused on developing parallel transfer techniques which can promote the throughput substantially by manipulating and placing multiple chips simultaneously in each transfer cycle. Stamp transfer printing, laser-assisted transfer, or fluid-assisted assembly are representative technologies with parallel transfer capability which are more worthy of further exploration than those techniques based on one-to-one transfer. The factors affecting the transfer yield and placement accuracy, however, must be extensively studied to suit the needs of volume production. Programmable transfer is another research subject to be looked at.

The capability for curved surface integration and 3D integration. Hetero-integration of various devices and materials onto curved surfaces or bendable substrates is highly desirable for demonstrating flexible optoelectronic devices or even 3D integrated devices [178,179]. While existing technologies such as stamp transfer printing can partially address this challenge, new layer-level and chip-level transfer techniques with improved throughput and better scalability are in high demand. The roll-to-roll transfer technology combined with specially design stamps, for instance, may be an elegant method for achieving this goal in future.
